# A Complex Network of Interactions between Mitotic Kinases, Phosphatases and ESCRT Proteins Regulates Septation and Membrane Trafficking in *S. pombe*

**DOI:** 10.1371/journal.pone.0111789

**Published:** 2014-10-30

**Authors:** Musab S. Bhutta, Brinta Roy, Gwyn W. Gould, Christopher J. McInerny

**Affiliations:** Henry Wellcome Laboratory of Cell Biology, Davidson Building, Institute of Molecular, Cell and Systems Biology, College of Medical, Veterinary and Life Sciences, University of Glasgow, Glasgow, United Kingdom; Kinki University School of Pharmaceutical Sciences, Japan

## Abstract

Cytokinesis and cell separation are critical events in the cell cycle. We show that Endosomal Sorting Complex Required for Transport (ESCRT) genes are required for cell separation in *Schizosaccharomyces pombe.* We identify genetic interactions between ESCRT proteins and polo and aurora kinases and Cdc14 phosphatase that manifest as impaired growth and exacerbated defects in septation, suggesting that the encoded proteins function together to control these processes. Furthermore, we observed defective endosomal sorting in mutants of *plo1*, *ark1* and *clp1*, as has been reported for ESCRT mutants, consistent with a role for these kinases in the control of ESCRT function in membrane traffic. Multiple observations indicate functional interplay between polo and ESCRT components: firstly, two-hybrid *in* *vivo* interactions are reported between Plo1p and Sst4p, Vps28p, Vps25p, Vps20p and Vps32p; secondly, co-immunoprecipitation of human homologues of Vps20p, Vps32p, Vps24p and Vps2p by human Plk1; and thirdly, *in* *vitro* phosphorylation of budding yeast Vps32p and Vps20p by polo kinase. Two-hybrid analyses also identified interactions between Ark1p and Vps20p and Vps32p, and Clp1p and Vps28p. These experiments indicate a network of interactions between ESCRT proteins, *plo1*, *ark1* and *clp1* that coordinate membrane trafficking and cell separation in fission yeast.

## Introduction

ESCRT proteins mediate membrane scission events involved in the down-regulation of ubiquitin-labelled receptors via the multivesicular body (MVB) pathway and in HIV budding from host cells [1]. In addition, ESCRT proteins play a role in abscission, the final stage of cytokinesis [2]. The ESCRT machinery is composed of four complexes: ESCRT-0, -I, -II and -III; and the modular composition of the ESCRT machinery is reflected in its various functions. For example, during MVB formation, ESCRT proteins are recruited sequentially to the endosomal membrane: ESCRT-0 sequesters ubiquitinated cargo destined for degradation, ESCRT-I and II deform the peripheral membrane to produce a bud and ESCRT-III constricts the bud neck to form an intralumenal vesicle [3]. Thereafter, the AAA-ATPase Vps4p redistributes ESCRT-III subunits into the cytoplasm to mediate further MVB formation; it is thought that the association of ESCRT-III and Vps4p forms the conserved membrane scission machinery in all ESCRT functions [4].

Similarly, at a precise time during cytokinesis, the ESCRT-I protein TSG101 and ESCRT-associated protein ALIX are recruited to the midbody through interactions with CEP55; TSG101 and ALIX in turn recruit ESCRT-III components [2]. Thereafter, by a mechanism still not completely understood, ESCRT-III redistributes to the putative abscission sites, microtubules are severed and the daughter cells separate. ESCRT-III then appears on the opposite side of the midbody and the process is repeated to produce the midbody remnant [5]–[7]. However, the mechanisms by which this selective and specific redistribution of ESCRT proteins is regulated in space and time remain largely unsolved.

ESCRT components are phosphoproteins [8] and more knowledge is being uncovered regarding regulation by kinases and phosphatases, such as polo and aurora kinases and Cdc14 phosphatase, due to their significant roles in controlling cytokinesis. Fission yeast aurora-related kinase Ark1p phosphorylates polo kinase Plo1p to drive mitotic commitment following recovery from nutrient-induced arrest [9]. Plo1p and the Cdc14-like phosphatase Clp1p are required for either formation or stabilisation of the contractile ring that drives cytoplasmic cleavage [3]. In mammalian cells, CEP55 depletion results in the failed accumulation of abscission factors at the midbody, including ESCRT proteins, Aurora B kinase and human polo-like kinase, Plk1, suggesting that these factors may be coordinately regulated [2]. Consistent with this, Aurora B phosphorylates the ESCRT-III component CHMP4C to delay abscission and prevent the accumulation of DNA damage [8]. Although Plk1 down-regulation is required for CEP55 midbody localisation, and thus ESCRT localisation, Plk1 reappears on midbodies late in telophase, where its function and targets remain unknown [10]. We therefore investigated whether polo and aurora kinases and Cdc14 phosphatase regulate members of the ESCRT machinery.

Here we show that ESCRT genes are required for cytokinesis and cell separation in fission yeast. An increased prevalence of septal defects was observed in double mutants of fission yeast strains containing individual ESCRT gene deletions, and a deletion mutant of *clp1* (*clp1Δ*) and temperature sensitive mutants of *plo1* and *ark1* (*plo1-ts35*, *ark1-T8* and *ark1-T11*). Consistent with the proposed interaction between ESCRT proteins and these key cytokinesis regulatory molecules, we observed synthetic growth defects in double mutants lacking individual ESCRT genes and mutant *plo1*, *ark1* and *clp1*. These interactions are supported by the observation that single mutants of *plo1*, *ark1* and *clp1* also affect vacuolar sorting, indicating novel roles for these proteins in ESCRT-dependent endosomal sorting processes. Yeast two-hybrid analyses revealed physical interactions between Plo1p and Sst4p (ESCRT-0), Vps28p (ESCRT-I), Vps25p (ESCRT-II), Vps20p and Vps32p (ESCRT-III). Interactions were also identified between Ark1p and both Vps20p and Vps32p, and Clp1p was shown to interact with Vps28p. Furthermore, we show that recombinant Plk1 can directly phosphorylate certain ESCRT components *in* *vitro*. Finally, co-immunoprecipitation experiments in mammalian cells suggest that interactions between ESCRT proteins and polo kinase are conserved across biological kingdoms.

In conclusion, we show that ESCRT proteins are involved in cytokinesis in *S. pombe*. Our studies reveal a network of genetic and protein interactions between the ESCRT machinery and polo and aurora kinases and Cdc14 phosphatase, some of which also occur in human cells; we further reveal a role for polo and aurora kinase in membrane trafficking to the yeast vacuole. These interactions likely coordinate membrane trafficking and cytokinesis, and hence characterising their functional significance will advance our understanding of the mechanisms that regulate these important aspects of cell biology.

## Materials and Methods

### Media and general techniques

General molecular procedures were performed [11], with standard methodology and media used for the manipulation of *S. pombe* [12]. The yeast strains used in this study are shown in Tables S4 and S5 in File S1. Cells were routinely cultured using liquid or solid YE medium at 25°C or 30°C. Over-expression of Ub-GFP-*Sp*CPS was from pREP41 [13], [14]. Cells were grown in EMM with 5 mg/ml thiamine (*nmt1* promoter ‘off’) to early exponential stage, washed three times in thiamine-free EMM and then grown for 16 hours in EMM without thiamine (*nmt1* promoter ‘on’).

### DNA constructs and RNA manipulations

The *plo1* ^+^, *mbx1* ^+^, *ark1^K147R^* and *clp1* ^+^ two-hybrid constructs have been described [15]–[18]. Wild-type *ark1* ^+^ was amplified by PCR from genomic DNA and ligated into the pBTM116 bait vector [18]. To create the other two-hybrid constructs, cDNA or genomic clones of *sst4^+^*, *sst6^+^*, *vps28^+^*, *vps36^+^*, *vps25^+^*, *vps20^+^*, *vps32^+^*, *vps2^+^* and *vps4^+^* were ligated into the pACT2 prey vector (CLONTECH). All constructs were confirmed by sequencing (University of Dundee). pREP41:Ub-GFP-*Sp*CPS has been described [14]. CHMP2A**-**GFP and CHMP6-GFP (Addgene plasmids 31805 and 31806, respectively) are described [6]; CHMP4B**-**YFP was a gift from J. Martin-Serrano [8], and CHMP3-myc was a gift from K. Bowers, University College London.

### Two-hybrid analysis

Two-hybrid analysis using *plo1* ^+^, *ark1* ^+^ and *clp1* ^+^ was carried out as described [15], [17], [18].

### Microscopy

*S. pombe* septa were visualised using Calcofluor [12], and vacuoles visualised with FM 4-64 (Invitrogen). Cells were placed in a chamber designed for liquid cultures (Lab-Tek II Chamber 155379), and confocal fluorescent microscopy performed using a Carl Zeiss LSM 5 Exciter microscope, fitted with a He/Ne and Ag laser system. Images were collected and processed using Microsoft PowerPoint. A two-tailed Student’s t-test was applied using Microsoft Excel. Individual phenotypes of single mutants were compared to the respective wild-type phenotype and individual phenotypes of double mutants were compared to the respective phenotype in each of their parent strains.

### Immunoprecipitation of Plk1 from HEK293 cell lysates

HEK293 cells were transfected with DNA for CHMP6-GFP, CHMP4B-YFP, CHMP3-myc or CHMP2A-GFP using Lipofectamine 2000 according to the manufacturer’s instructions. One mg of cell lysate was incubated overnight at 4°C with rotation with 12.8 µg anti-Plk1 antibody. After incubation, 30 µl Protein A sepharose beads (GE Healthcare Life Sciences), pre-washed twice and resuspended in cold lysis buffer, was added and lysate mixtures were again incubated with rotation at 4°C for two hours. After incubation, mixtures were centrifuged briefly at 4°C and the supernatant was retained. Beads were washed twice in IP wash buffer 1 (20 mM Hepes, 100 mM KCl and 1 mM DTT) and thrice in IP wash buffer 2 (20 mM Hepes, 250 mM NaCl, 1% Triton X-100 and 1 mM DTT). Beads were then resuspended in 75 µl Laemmli sample buffer, mixed vigorously using a vortex and heated to 65°C for ten minutes. Beads were centrifuged for five minutes and the supernatant was retained.

### Antibodies

For immunoblotting, all antibodies were incubated overnight at 4°C. Anti-Plk1 antibody, Cell Signaling 208G4; anti-GFP, Abcam ab290; anti-myc, Abcam ab9106.

### Recombinant protein expression and kinase assays

Plasmids encoding budding yeast Vps20p, Snf7p or Vps2p (Addgene plasmids 21494, 21492 and 21490, respectively) [4] were transformed into *E. coli* BL21 (DE3) cells and expression of the MBP-His-fusion proteins induced as described [4]. Proteins were purified using Ni-agarose and *in* *vitro* kinase assays were performed as follows. 2 µg of recombinant protein was mixed in assay buffer (25 mM MOPS, 25 mM MgCl_2_, 1 mM EDTA, 0.25 mM DTT) and 125 ng of recombinant Plk1 (activity 32 U/mg; Millipore, Dundee) was added. Kinase reactions were initiated by the addition of ATP such that the final concentration was 100 µM ATP/6 µCi [^32^P-γ]-ATP. Samples were incubated at 30**°**C and the reactions stopped by the addition of Laemmli sample buffer. Samples were heated to 65**°**C and separated by SDS-PAGE. Phosphorylation events were assayed by autoradiography, excised where necessary and quantified using Cerenkov counting.

## Results

### ESCRT mutants in fission yeast display cell division defects

There is increasing evidence that ESCRT proteins play a role during cytokinesis and abscission in eukaryotic cells [2], [8], [19]. To test whether ESCRT proteins are involved in cell separation in fission yeast, we examined the effect of chromosomal deletions of individual genes encoding the various classes of ESCRT proteins on cytokinesis and septation.

**Figure 1:**
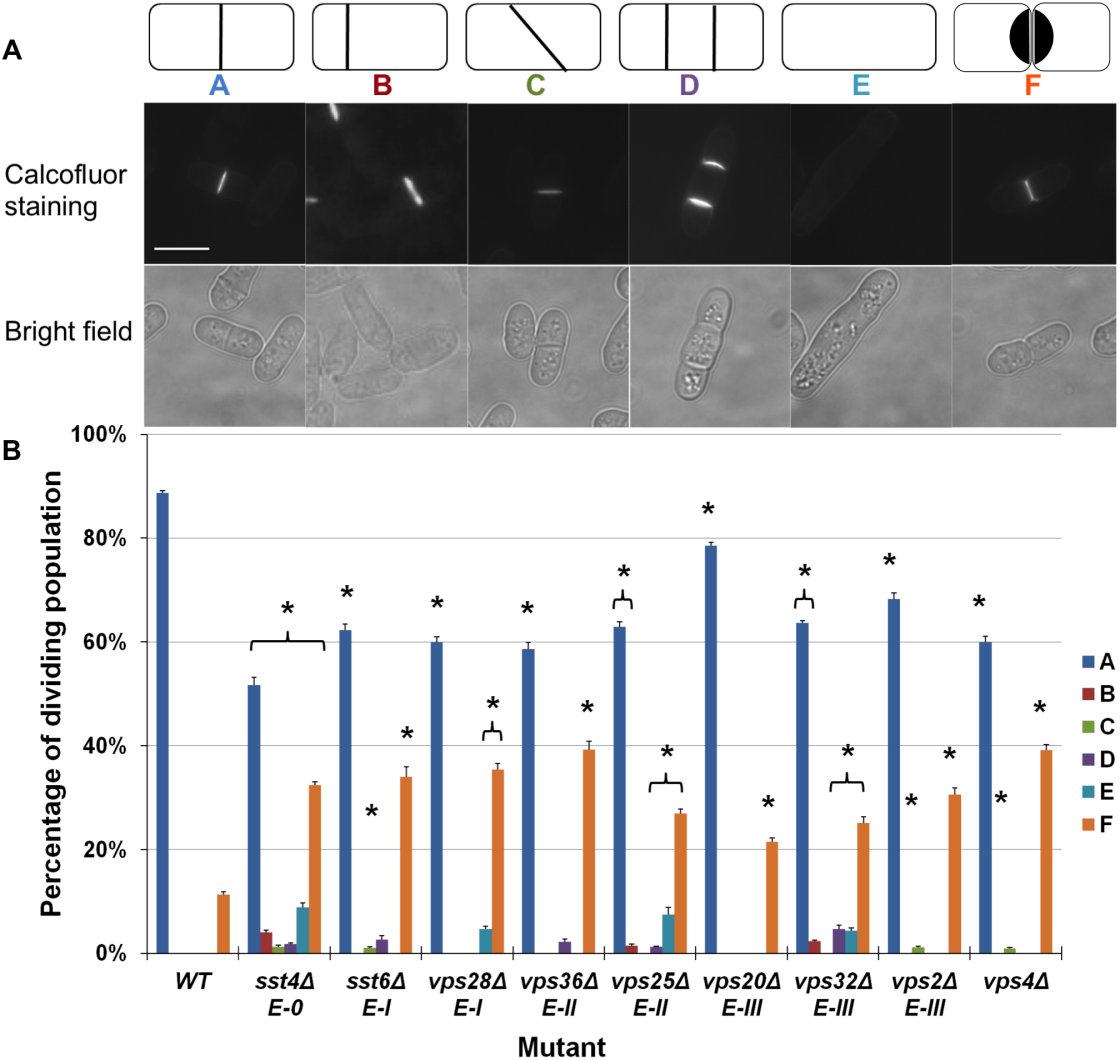
ESCRT proteins are required for septation in fission yeast. (a) Defective septation in fission yeast strains containing chromosomal deletions of ESCRT genes. Wild-type and strains containing individual chromosomal deletions of ESCRT genes were grown at 25°C in complete liquid medium to mid-exponential phase and harvested. Cells were stained with Calcofluor white and visualised using fluorescence microscopy. Both fluorescence and bright field images are shown. Panels A-F show representative cells illustrating observed septation phenotypes. Schematic diagrams above panels represent each phenotype: (A) a normal septum, (B) a misaligned septum, (C) a non-perpendicular septum, (D) multiple septa, (E) no septal formation, and (F) failed separation of daughter cells following septation. Scale bars, 10 µm. Data from a typical experiment repeated three times is shown. (b) Quantitative analysis of the frequency of septation phenotypes A–F in strains containing ESCRT chromosomal deletions, in comparison to wild-type. In each case, 400 cells were counted in triplicate. An asterisk (*) indicates a *p* value<0.05, indicating a significant difference to wild-type; n = 3. Each of the ESCRT gene labels is accompanied by its respective ESCRT complex identification (*E-0*, *E-I*, *E-II* and *E-III*).

Microscopic visualisation of dividing cells in wild-type and ESCRT deletion mutant strains revealed striking differences in their respective septation indices (Fig. 1). An asynchronously dividing population of wild-type cells displayed ∼87% containing normal septa (Class A) and ∼12% separating cells (Class F). Deletion mutants of genes encoding components of the various classes of ESCRT proteins were viable [14]. Significantly different numbers of cells showing the Class F phenotype were displayed in *sst4Δ* (∼42%, ESCRT-0), *vps20Δ* (∼46%), *vps24Δ* (∼45%, ESCRT-III) and *vps4Δ* (∼45%) cells, suggesting that the timing of separation in these cells was affected (Fig. 1b). Furthermore, *sst4Δ* (ESCRT-0), *sst6Δ* and *vps28Δ* (ESCRT-I), *vps25Δ* (ESCRT-II), *vps20Δ*, *vps24Δ* and *vps2Δ* (ESCRT-III), and *vps4Δ* cells displayed additional defective septal phenotypes (Classes B–D), suggesting that septal formation had occurred incorrectly. Some mutants also showed the absence of septa in cells of a length that would be expected to contain them (Class E), such as *sst6Δ*, *vps28Δ*, *vps25Δ*, *vps24Δ* and *vps4Δ*, which suggested that septal formation was delayed. Similar observations were made at 30°C (Fig. S1). Collectively, these observations demonstrate that septal formation is affected by the absence of various classes of ESCRT proteins in fission yeast, implying that they contribute to cell separation in this organism.

### Genetic interactions between mutants of ESCRT, polo and aurora kinases, and Cdc14 phosphatase

To identify potential regulators of ESCRT proteins, a genetic approach was adopted. Polo and aurora kinases and Cdc14 phosphatases were chosen on the basis of their prominent roles regulating late cell cycle events in eukaryotic organisms. Yeast strains containing chromosomal deletions of the various classes of ESCRT proteins were crossed with mutants of polo (*plo1-ts35*, a temperature-sensitive lethal strain) [20], aurora (*ark1-T8* and *ark1-T11*, both temperature-sensitive lethal strains) [21] and Cdc14 (*clp1Δ* chromosomal deletion [22], *clp1.D257A* phosphatase-dead, and *clp1.3A* a gain-of-function mutant with reduced phosphorylation by Cdc2p) [23] to search for synthetic phenotypes.

These crosses resulted in viable progeny in all cases, but synthetic growth defects in double mutants compared with single mutants were consistently observed (Fig. S2 and Table S1 in File S1), which suggested genetic interactions. We detected genetic interactions between *plo1* ^+^ and *sst4^+^*, *sst6^+^*, *vps28^+^*, *vps25^+^*, *vps20^+^*, *vps2^+^* and *vps4^+^*, but not *vps36^+^*. Further, we found that *ark1* ^+^ interacted selectively with *sst4^+^*, *vps36^+^*, *vps20^+^* and *vps4^+^*. The observed interactions with *clp1* ^+^ varied according to the mutant examined: *clp1Δ* showed synthetic phenotypes with all ESCRT mutants apart from *vps25^+^*; whereas *clp1.D257A* showed synthetic phenotypes with just *sst4^+^*, *vps20^+^* and *vps4^+^*; and *clp1.3A* showed synthetic phenotypes with *vps28^+^*, *vps36^+^*, *vps20^+^* and *vps4^+^*.

### Cell division defects in fission yeast ESCRT deletion strains are exacerbated in *plo1*, *ark1* and *clp1* mutants

**Figure 2:**
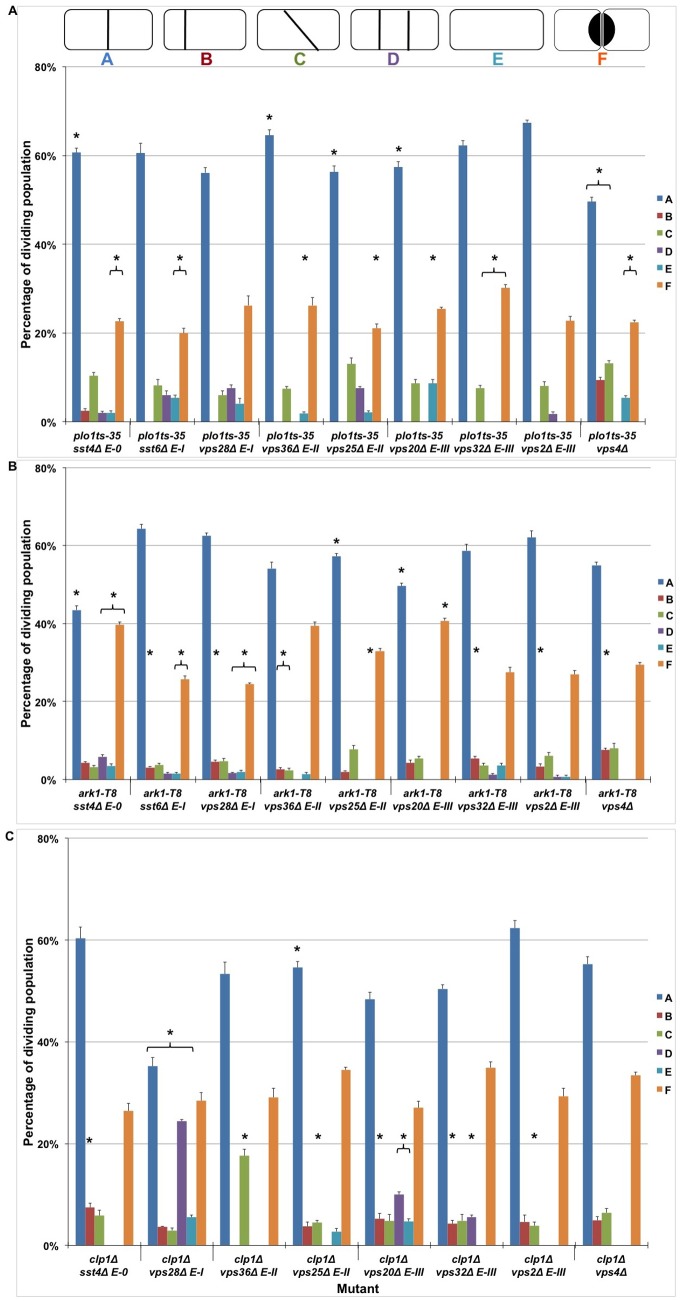
Synthetic septation phenotypes observed in double mutants in genes encoding ESCRT proteins and (a) *plo1-ts35*, (b) *ark1-T8* or (c) *clp1Δ*. Fission yeast double mutant strains were grown in complete liquid medium at 25°C to mid-exponential phase and harvested. Cells were stained with Calcofluor white and visualised using fluorescence microscopy. Both fluorescence and bright field images are shown. Scale bar, 10 µm. The frequency of phenotypes A–F (described in Figure 1a) was quantitatively analysed in strains containing double mutants, in comparison to each parent. In each case 400 cells were counted in triplicate (*p<0.05). Each of the ESCRT genes labels is accompanied by its respective ESCRT complex identification (*E-0*, *E-I*, *E-II* and *E-III*).

To further understand the relationship between ESCRT proteins and polo and aurora kinases and Cdc14 phosphatase, double mutants were examined for potential septal defects. Double mutants were compared to both sets of respective mutant parents, and the septation profiles for *plo1-ts35*, *clp1Δ*, *ark1-T8* and *ark1-T11* single mutants relative to wild-type have been shown in Fig. S3. A higher proportion of phenotype Classes C–F was observed in *plo1-ts35* cells, and of Classes C and F in *clp1Δ* cells (Fig. S3). Interestingly, *plo1* mutants when combined with individual mutations in ESCRT genes frequently revealed a decreased proportion of the Class F phenotype, indicative of phenotypic rescue and the interaction of these genes within a shared pathway. For instance, the *vps4Δ* strain exhibited the Class F phenotype in 39% of cells; this was reduced to 23% in *plo1-ts35 vps4Δ* cells (*p*<0.05 relative to both parents; Fig. 2a). Both sets of double mutants also exhibited increases in phenotype Classes B–E (Fig. 2a and 2c). An increased proportion of Class B, E and F was observed in *ark1-T8* cells (Fig. S3), and analysis of double mutants revealed a higher prevalence of Classes B in particular (Fig. 2b). A similar increase in Class B was observed in double mutants with *ark1-T11* (Fig. S4). Collectively, these data are highly suggestive of genetic interactions between ESCRT and *plo1*, *ark1* and *cdc14* genes, and imply cooperation of these genes to control cell separation in this organism.

### *In* *vivo* interactions between ESCRT proteins and Plo1p, Ark1p and Clp1p

**Figure 3:**
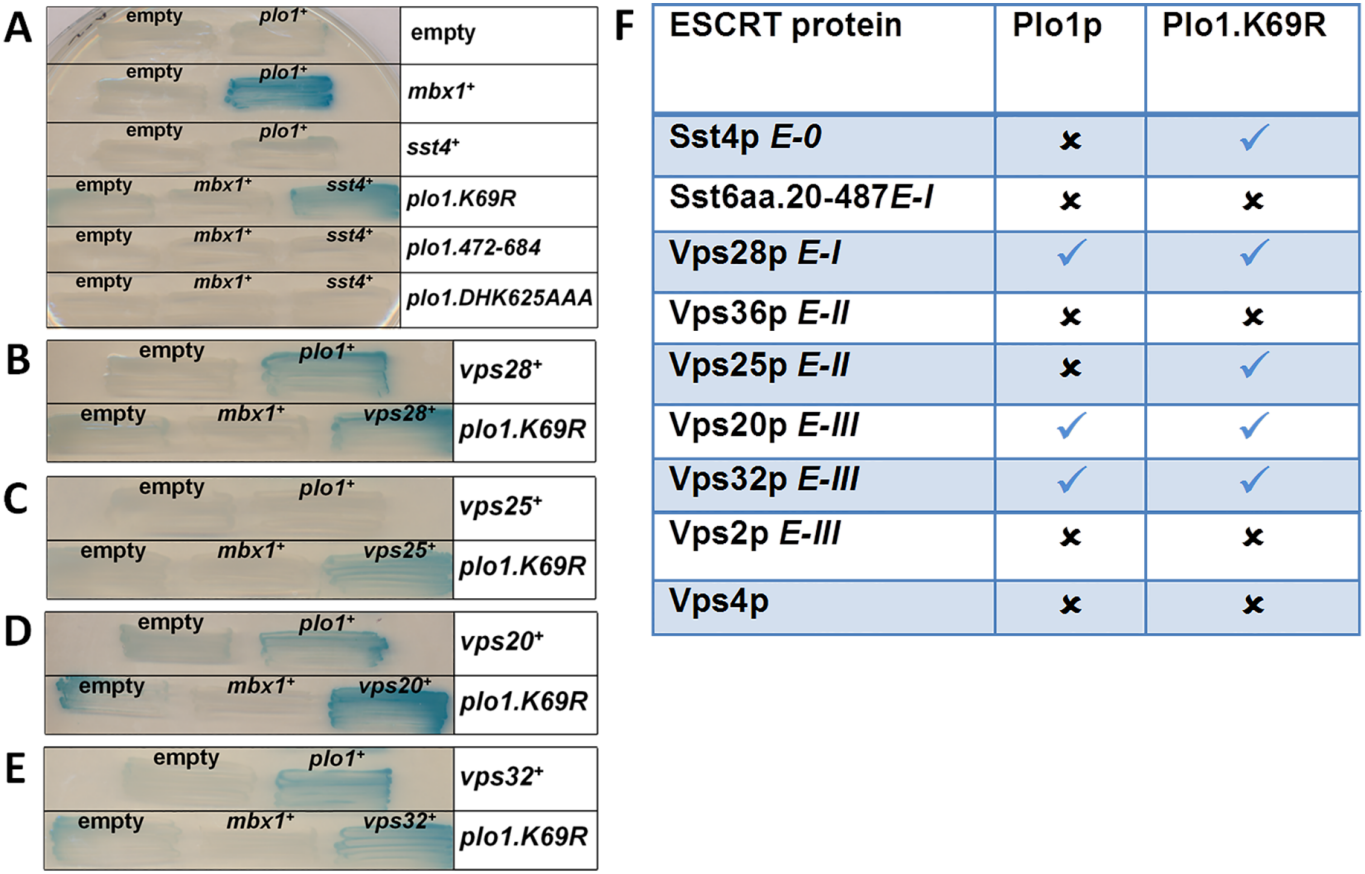
Physical interactions between ESCRT proteins and Plo1p as revealed by two-hybrid analysis. (a–e) Budding yeast strains containing *LEXA*–galactosidase transcriptional readout were transformed with the yeast two-hybrid bait (LexA DNA binding domain) and prey (GAL4 transcription activation domain) constructs indicated. Plo1p was fused to LexA and ESCRT proteins from each class were fused to GAL4. Mbx1p, a known Plo1p interacting protein [17], was used a positive control. Interactions with mutants in Plo1p in the kinase domain (*K69R*) and polo boxes (*472–684* and *DHK625AAA*) were also performed [18]. Transformed strains were grown for three days on selective medium with the X-gal overlay assay then performed. Experiments were performed three times with qualitatively similar results and illustrative examples of performed two-hybrid reactions are shown. (f) Summary of physical interactions between Plo1p and ESCRT proteins identified by yeast two-hybrid analysis. The table indicates the presence or absence of yeast showing a blue colour observed when each of the ESCRT proteins with various versions of Plo1p was assayed.

**Figure 4:**
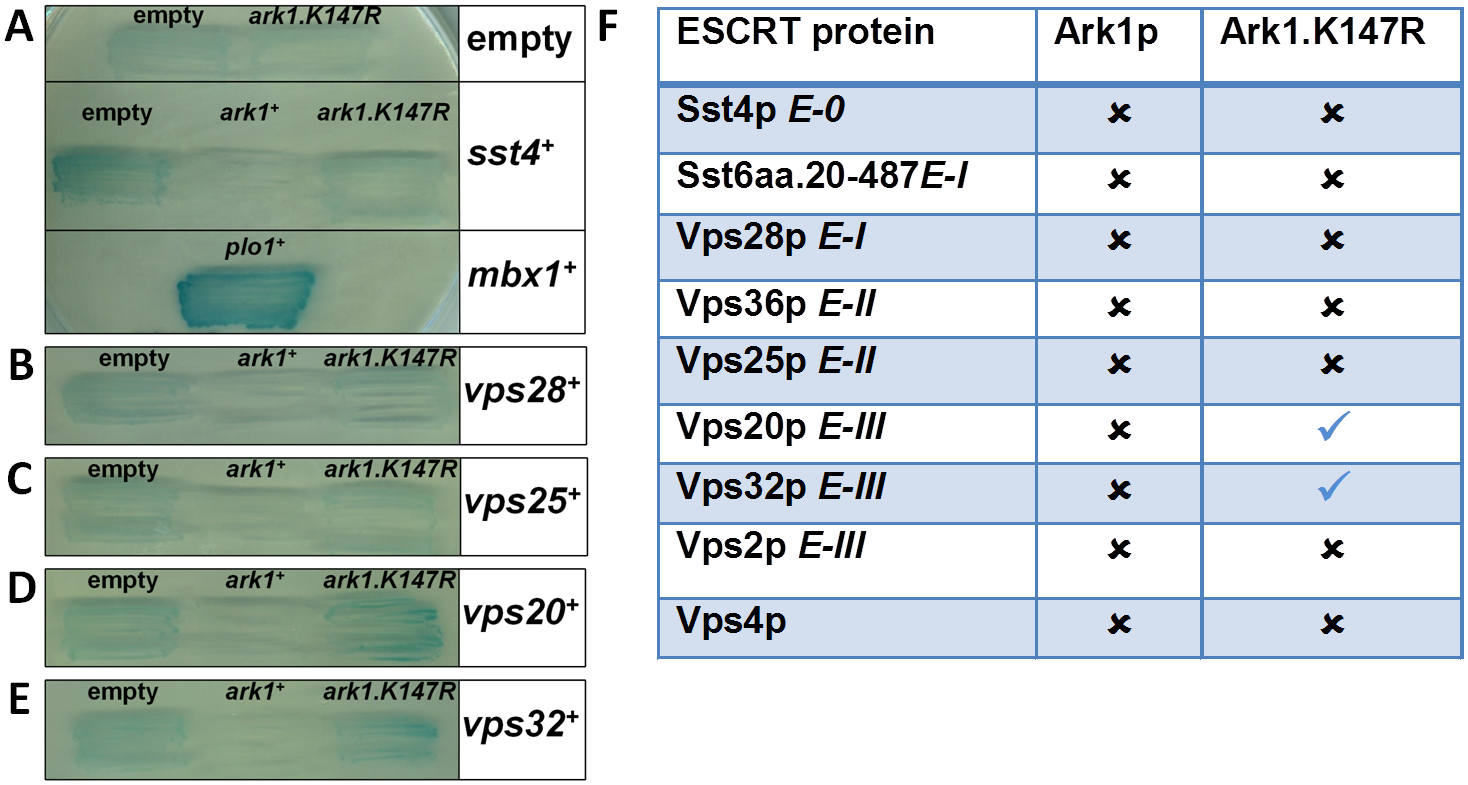
Physical interactions between ESCRT proteins and Ark1p as revealed by two-hybrid analysis. (a–e) Budding yeast strains containing *LEXA*–galactosidase transcriptional readout were transformed with the yeast two-hybrid bait (LexA DNA binding domain) and prey (GAL4 transcription activation domain) constructs indicated. Ark1p was fused to LexA and ESCRT proteins from each class were fused to GAL4. Mbx1p, a known Plo1p interacting protein,^28^ was used a positive control for the assay. Transformed strains were grown for three days on selective medium with the X-gal overlay assay then performed. Experiments were performed three times with qualitatively similar results and illustrative examples of performed two-hybrid reactions are shown. (f) Summary of physical interactions between Ark1p and ESCRT proteins identified by yeast two-hybrid analysis. The table indicates the presence or absence of yeast showing a blue colour observed when each of the ESCRT proteins with various versions of Ark1p was assayed.

**Figure 5:**
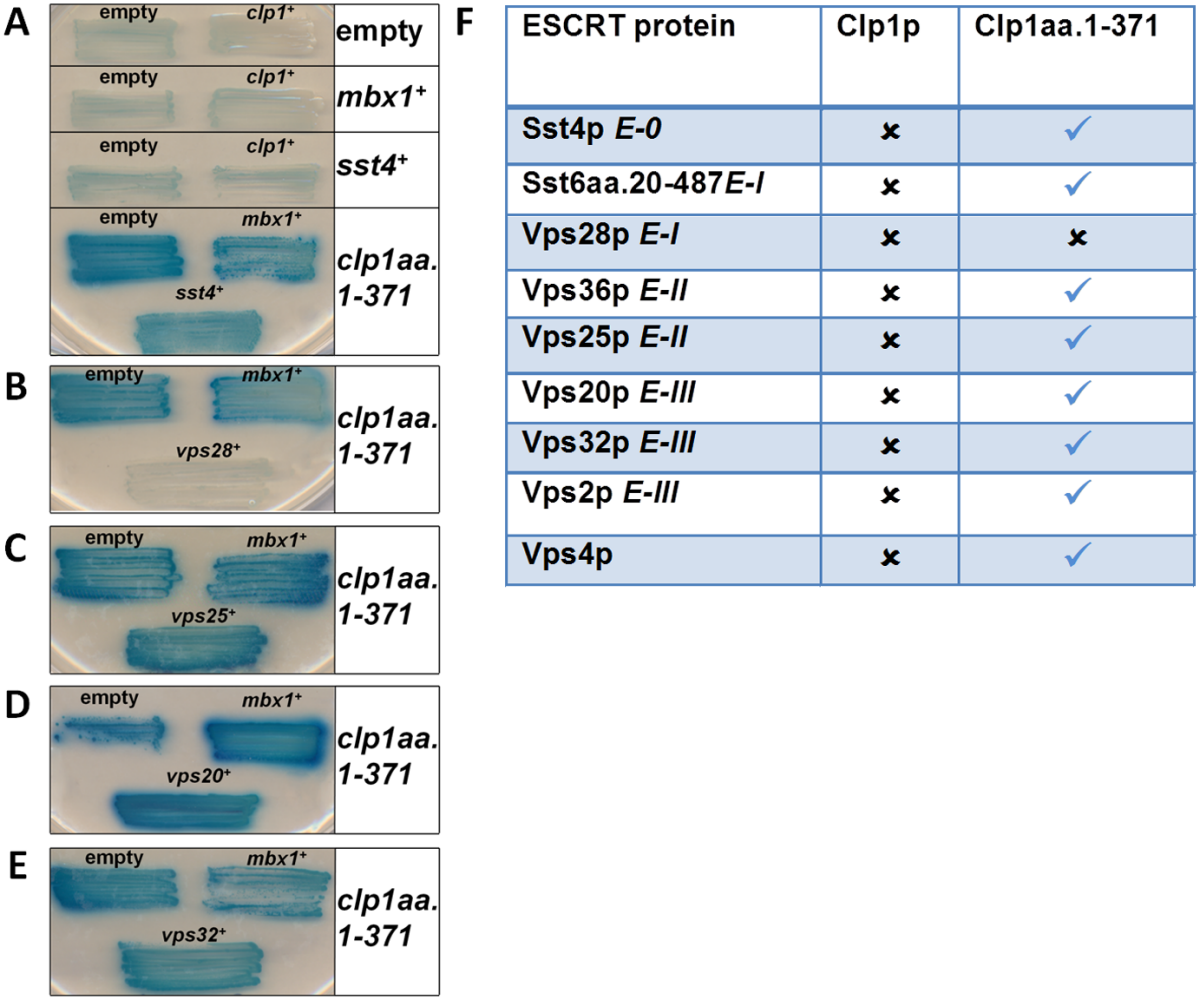
Physical interactions between ESCRT proteins and Clp1p as revealed by two-hybrid analysis. (a–e) Budding yeast strains containing *GAL4*-galactosidase transcriptional readout were transformed with the yeast two-hybrid bait (GAL4 DNA binding domain) and prey (GAL4 transcription activation domain) constructs indicated. Clp1p was fused to GAL4 and ESCRT proteins from each class were fused to GAL4. Mbx1p, a known Clp1p interacting protein [17], was used a positive control for the assay. Transformed strains were grown for three days on selective medium with the X-gal overlay assay then performed. Experiments were performed three times with qualitatively similar results and illustrative examples of performed two-hybrid reactions are shown. (f) Summary of physical interactions between Clp1p and ESCRT proteins identified by yeast two-hybrid analysis. The table indicates the presence or absence of yeast showing a blue colour observed when each of the ESCRT proteins with Clp1p was assayed.

One explanation for the observed genetic interactions is that the encoded proteins directly interact. We therefore used two-hybrid analysis to qualitatively search for *in* *vivo* interactions between ESCRT proteins and Plo1p, Ark1p and Clp1p. A panel of ESCRT proteins was screened using Plo1p (wild-type, kinase-dead and polo box mutants (Fig. 3)), Ark1p (wild-type and a kinase-dead mutant (Fig. 4)) and Clp1 (wild-type and a truncation mutant (Fig. 5)). These analyses revealed that Plo1p interacts with Sst4p, Vps28p, Vps25p, Vps20p and Vps32p, but not Sst6p, Vps36p, Vps2p or Vps4p (Fig. 3). We found Ark1p interacted only in a kinase-inactivated form with Vps20p and Vps32p (Fig. 4); this is evident on qualitatively comparing Ark1.K147R to the empty vector. Clp1p was observed to interact only with Vps28p (Fig. 5); this was noted on the basis of the lack of transcriptional readout, which we propose to occur via Vps28p-mediated sequestration of Clp1aa.1-371 from transcriptional auto-activation.

### Endosomal sorting defects in *plo1*, *ark1* and *clp1* mutants

**Figure 6:**
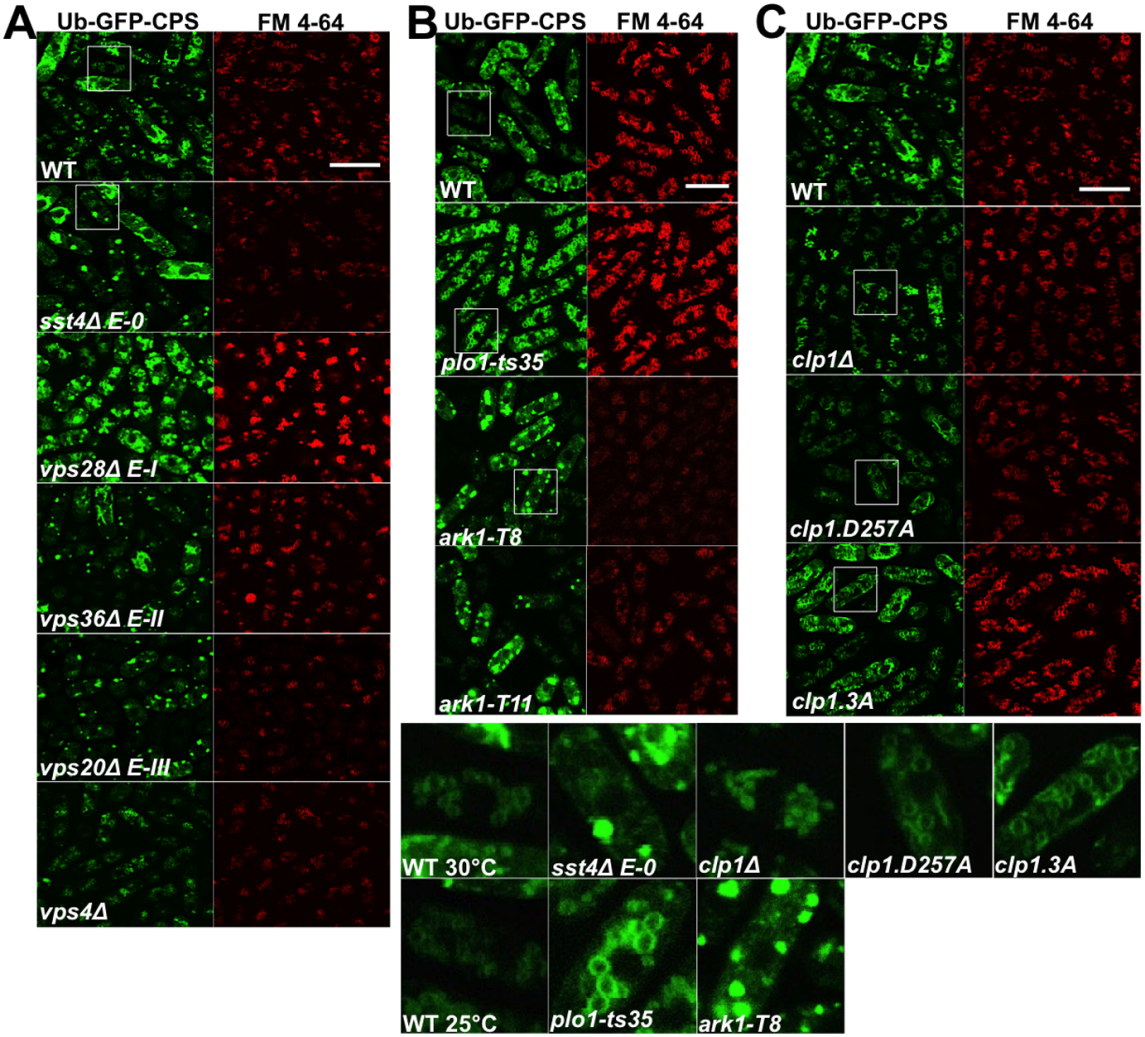
ESCRT proteins and Plo1p, Ark1p and Clp1p are required for vacuolar sorting in fission yeast. (a) Defective vacuolar sorting is observed in fission yeast with individual chromosomal deletions of ESCRT genes. Wild-type and ESCRT-deleted fission yeast strains, transformed with Ub-GFP-*Sp*CPS, were cultured in liquid minimal medium at 25°C, stained with FM 4-64, and visualised using confocal microscopy. Mutants of *plo1* and *ark1* (b) and *clp1* (c) cause defective vacuolar sorting in fission yeast. Wild-type and fission yeast *plo1-ts35*, *ark1-T8*, *ark1-T11* and *clp1* strains, transformed with Ub-GFP-*Sp*CPS, were cultured in liquid minimal medium, stained with FM 4-64, and visualised using a confocal microscope. Strains were cultured at 25°C or 30°C. Scale bar, 10 µm. Experiments were performed three times with qualitatively similar results and data from a typical experiment are shown.

Various classes of ESCRT proteins function as part of the endosomal sorting machinery [24], [25], and ESCRT genes have been shown to be required for correct Ub-GFP-*Sp*CPS localisation in fission yeast [14]. The distribution of Ub-GFP-*Sp*CPS in cells containing mutants of *plo1*, *ark1* and *clp1* was examined. Consistent with published data, Ub-GFP-*Sp*CPS expressed in wild-type cells exhibited a largely vacuolar pattern of sorting, identified by the fluorescent profiles of Ub-GFP-*Sp*CPS and FM 4-64-stained vacuoles, which strongly suggest co-localisation (Fig. 6a) [14]. However, yeast strains containing ESCRT deletions, with one mutant chosen from each ESCRT class, exhibited a punctate pattern of Ub-GFP-*Sp*CPS fluorescence associated with endosomal staining (Fig. 6a), as reported by Iwaki et al [14].

When strains with mutations in *plo1*, *ark1* or *clp1* were examined for Ub-GFP-*Sp*CPS localisation, distinct vacuolar profiles were noted. *plo1-ts35* cells exhibited a uniform array of vacuoles that appear to co-localise with Ub-GFP-*Sp*CPS (Fig. 6b). In contrast, *ark1-T8* and *ark1-T11* both showed a punctate pattern of fluorescence similar to that observed in strains containing ESCRT deletions (Fig. 6b). *clp1* mutants showed various phenotypes (Fig. 6c): Ub-GFP-*Sp*CPS predominately localised to the lumen of *clp1Δ* cells, whereas *clp1.D257A* cells revealed a phenotype closer to wild-type; and *clp1.3A* cells revealed a phenotype similar to that observed in *plo1-ts35* cells.

### Epistasis of endosomal sorting defects in ESCRT, *plo1*, *ark1* and *clp1* mutants

These observations prompted an epistasis experiment, whereby the Ub-GFP-*Sp*CPS construct was transformed into double mutants of ESCRT deletions and *plo1-ts35*, *ark1-T8* and *clp1Δ*; one representative of each ESCRT class was selected. This allowed the qualitative analysis of the Ub-GFP-*Sp*CPS and vacuolar distributions in double mutants compared to each parent to allow inferences about the dependency of ESCRT proteins on Plo1p, Ark1p and Clp1p to control the cell sorting function.

**Figure 7:**
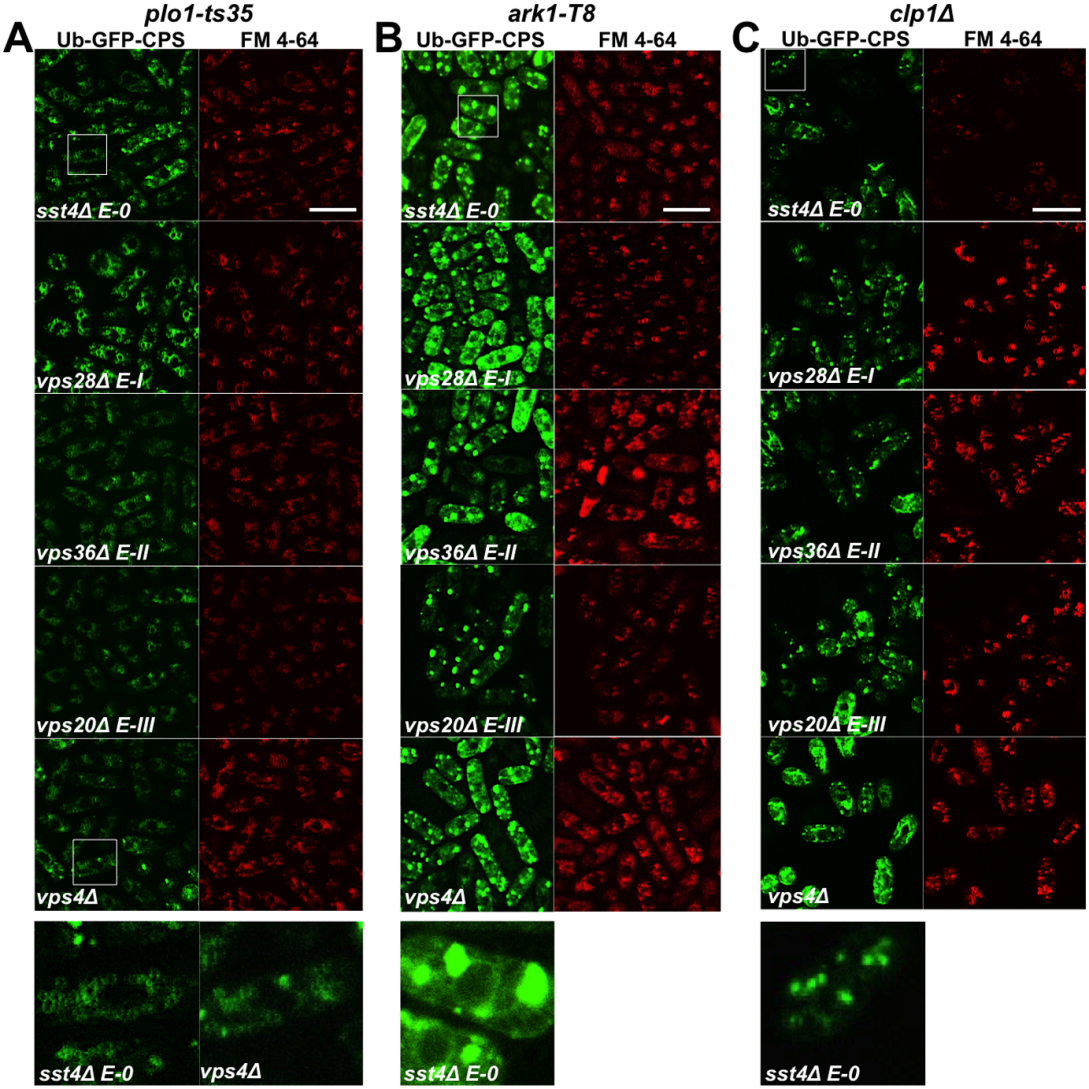
Genetic interactions between *plo1-ts35, ark1-T8* and *clp1Δ,* and ESCRT deletions in controlling vacuolar cell sorting in fission yeast. Double mutants of ESCRT deletions and (a) *plo1-ts35*, (b) *ark1-T8* or (c) *clp1Δ*, transformed with Ub-GFP-*Sp*CPS, were cultured in liquid minimal medium at 25°C, stained with FM 4-64, and visualised using confocal microscopy. Scale bar, 10 µm.

All but one of the *plo1-ts35* ESCRT double mutants, *plo1-ts35 vps4Δ*, exhibited a phenotype closer to their *plo1-ts35* parent (Fig. 7a). The *ark1-T8* ESCRT double mutants were indistinguishable from both parents (Fig. 7b). In contrast, all of the *clp1Δ* ESCRT double mutants exhibited a phenotype closer to their ESCRT parent (Fig. 7c). The results of these experiments are summarised in Table S2 in File S1. These epistasis data suggest that *plo1^+^* functions upstream of the ESCRT genes, and *clp1^+^* downstream, in controlling endosomal sorting in fission yeast.

### Human polo-like kinase 1 (Plk1) interacts with ESCRT-III proteins in mammalian cells

**Figure 8:**
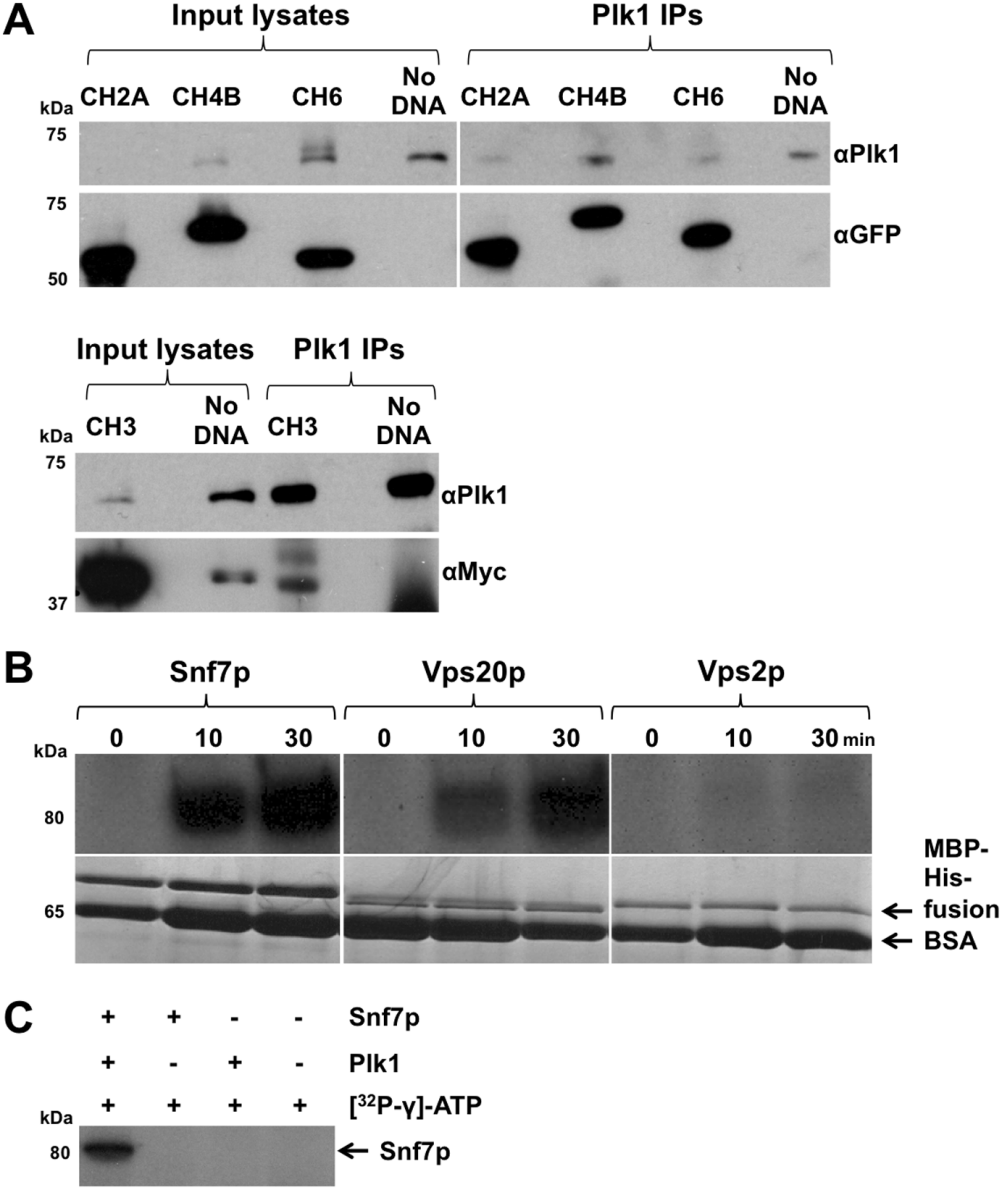
Co-immunoprecipitation and *in* *vitro* phosphorylation of ESCRT proteins by human Plk1. (a) HEK293 cells were transfected with DNA for CHMP6-GFP, CHMP4B-YFP, CHMP3-myc, CHMP2A-GFP, or with Lipofectamine 2000 in the absence of transforming DNA. Lysates were prepared and anti-Plk1 antibody was added to each cell lysate. Complexes were dissociated from beads (*IP*: one-eighth was loaded). *Input* refers to the cell lysate that was interrogated: 10 µg was loaded. Experiments were repeated with qualitatively similar results. (b) MBP-His-ESCRT proteins were purified from *E. coli* and incubated at 30°C with recombinant Plk1 and [^32^P-γ]-ATP for 0, 10 and 30 minutes. The reaction was stopped by the addition of Laemmli sample buffer. Samples were heated to 65°C, separated by SDS-PAGE and assayed by autoradiography. Shown is a typical experiment repeated three times using two different preparations of each recombinant protein and two different batches of recombinant Plk1. Coomassie Blue was used to confirm equal loading of recombinant ESCRT in each of the lanes. The major band at 65 kDa is BSA from the buffer used to resuspend Plk1. (c) MBP-His-Snf7p purified from *E. coli* and incubated at 30°C with or without recombinant Plk1 and [^32^P-γ]-ATP for 30 minutes. Molecular weight markers are indicated.

To test whether the interactions observed using two-hybrid assays are conserved in humans, immunoprecipitation/co-immunoprecipitation assays were performed. Human ESCRT proteins tagged with GFP or myc were over-expressed in HEK293 cells and Plk1 was immunoprecipitated from lysates. SDS-PAGE and immunoblotting revealed co-immunoprecipitation of CHMP6 (fission yeast Vps20p), CHMP4B (Vps32p), CHMP3 (Vps24p) and CHMP2A (Vps2p) (Fig. 8a). Separate experiments were performed to confirm these interactions. Firstly, CHMP6-GFP, but not FIP3-GFP, was shown to co-immunoprecipitate with Plk1 (Fig. S5a). Secondly, sequential immunoprecipitation using anti-Plk1 antibody depleted Plk1 levels in HEK293 cell lysates, as well as those of CHMP6 (Fig. S5b). However, CHMP6 was detected in the unbound input following the third anti-Plk1 immunoprecipitation at qualitatively comparable levels to the first input. Subsequent incubation with Protein A sepharose beads in the absence of antibody did not result in co-immunoprecipitation of CHMP6 despite the high protein levels detected in the final unbound input. These data indicate that ESCRT proteins are detected in anti-Plk1 immunoprecipitations due to specific interactions with Plk1. Thus, polo kinase physically interacts with ESCRT proteins in both fission yeast and human cells.

### Plk1 phosphorylates ESCRT proteins

One potential way that polo kinase could regulate ESCRT function is through direct phosphorylation. To test this possibility we examined the ability of recombinant human Plk1 to phosphorylate recombinant ESCRT proteins purified from bacteria. Specifically, we examined ESCRT proteins whose interactions with polo kinase were previously characterised through two-hybrid analyses. Budding yeast Snf7p (fission yeast Vps32p), Vps20p and Vps2p were purified from *Escherichia coli* and incubated at 30°C with Plk1 and [^32^P-γ]-ATP for 0, 10 and 30 minutes. SDS-PAGE and autoradiography revealed phosphorylation by Plk1 of Snf7p and Vps20p, but not Vps2p (Fig. 8b). Furthermore, phosphorylation of Snf7p was shown to occur only in the presence of both recombinant Plk1 and [^32^P-γ]-ATP (Fig. 8c). These data confirm that these ESCRT proteins are *in* *vitro* substrates of Plk1.

## Discussion

Considerable evidence has accumulated supporting a role for ESCRT proteins in abscission in mammalian cells. Together with a role for ESCRT proteins in cell division in Archaea [26], this suggests a conserved abscission mechanism across phyla. In budding yeast however, ESCRT proteins appear to play a less important role in cytokinesis, with their contributions revealed when studied in tandem with other genetic deletions [27]. Here we show for the first time that ESCRT genes are involved in cell division in fission yeast. Staining newly deposited cell wall facilitated characterisation of a number of septation phenotypes in fission yeast with individual chromosomal deletions of ESCRT genes (Fig. 1). The Class F phenotype, characterised as delayed separation following septation, has been described in wild-type cells as ‘hanging’ following division for 0.12 of a cell cycle [28]. The Class F phenotype described here was noted due to its significantly (*p*<0.05) increased occurrence among cells with ESCRT gene deletions. It was on this basis that it was identified as defective cell separation. Thus, we conclude that fission yeast utilises ESCRT proteins in cell separation, but unlike their budding yeast relatives, this role does not appear to be necessary for cell division to be successfully completed.

We reasoned that these separation defects may facilitate an analysis of control pathways regulating ESCRT function. To that end, fission yeast double mutants were generated by crossing strains containing individual deletions of ESCRT genes with temperature-sensitive mutant strains *plo1-ts35*, *ark1-T8* and *ark1-T11*, along with various mutants of *clp1*. All double mutants in this study were found to be viable but subtle synthetic interactions were inferred by assaying growth rate between parent and offspring strains (Fig. S2). This principle has been shown previously between *plo1* ^+^ and *clp1* ^+^, whereby *clp1.D257A* reduced the restrictive temperature of *plo1-ts35*, indicative of exacerbated cell stress as a result of genetic interactions [29]. Defective growth rate was observed among double mutants with *plo1*, *ark1* and *clp1* from every class of ESCRT gene (0, I, II and III). Furthermore, an increased prevalence of septal defects was observed in double mutants of fission yeast strains with individual ESCRT gene deletions and *plo1-ts35*, *ark1-T8*, *ark1-T11* and *clp1Δ* (Fig. 2, and Table S1 in in File S1 and Fig. 3b).

Genetic interactions between *plo1* ^+^, *ark1* ^+^, *clp1* ^+^ and ESCRT genes were further confirmed by studies of Ub-GFP-*Sp*CPS trafficking. The ESCRT machinery is required for downregulation of ubiquitin-labelled receptors via the multivesicular body pathway, and the requirement of individual ESCRT genes for correct sorting has been demonstrated (Fig. 6a) [14]. Fission yeast strains with mutations in *plo1* and *clp1* showed defective sorting, but with distinct phenotypes to that observed in ESCRT deletion strains; in contrast cells with mutants in *ark1* showed similar phenotypes to ESCRT mutants (Fig. 6b and 6c). These data reveal novel functions for *plo1* ^+^, *ark1* ^+^ and *clp1* ^+^ in vacuolar sorting in fission yeast. To investigate the dependency of ESCRT proteins on Plo1p, Ark1p and Clp1p in controlling vacuolar sorting, double mutants of *plo1-ts35*, *ark1-T8* and *clp1Δ* with ESCRT deletions were examined. Double mutant phenotypes were more similar to *plo1-ts35*, although the converse was noted for *plo1-ts35 vps4Δ* (Fig. 7a). Analysis of double mutants of *clp1Δ* and individual ESCRT deletions revealed phenotypes more similar to each respective ESCRT single deletion mutant (Fig. 7c). These epistasis experiments suggest that *plo1^+^* functions upstream of the ESCRT genes in controlling vacuolar sorting, and that *clp1* ^+^ functions downstream of the ESCRT genes. These data are consistent with a model of regulation whereby Plo1p and Clp1p interact with ESCRT proteins, respectively, before and after ESCRT functions in vacuolar sorting. In particular, the similarity between the *plo1-ts35* and *clp1.3A* mutant strains may indicate that increased Clp1p activity conferred by this gain-of-function mutation reverses Plo1p phosphorylation to maintain ESCRT proteins in a hypophosphorylated form, thus trapping Ub-GFP-*Sp*CPS upon the peripheral membrane. The genetic dependency of ESCRT genes upon *plo1* ^+^, *ark1^+^* and *clp1* ^+^ may be further characterised by producing yeast strains with multiple deletions in ESCRT genes. Epistasis experiments of this type may allow the specific genetic pathway involving these genes in vacuolar sorting to be identified; this may resolve the genetic interactions between *ark1^+^* and the ESCRT genes, whose sorting phenotypes were identical. Our data are also consistent with a model in which the role of ESCRT proteins in budding yeast cytokinesis was ascribed to the control of membrane trafficking [27].

Using two-hybrid analyses, we have shown that Plo1p physically interacts with proteins in all ESCRT classes, 0, I, II and III. We have also shown physical interactions between Ark1p and Vps20p and Vps32p (ESCRT-III), as well as between Clp1p and Vps28p (ESCRT-I). Such observations suggest that Plo1p may regulate ESCRT proteins involved in processes such as vacuolar sorting and cytokinesis. The implication of these data is that Plo1p, Ark1p and Clp1p regulate ESCRT proteins via their phosphorylation state. In support of this hypothesis, we have shown that recombinant human Plk1 can selectively phosphorylate recombinant ESCRT-III proteins, specifically Vps20p and Snf7p (fission yeast Vps32p). This phosphorylation is selective, as Vps2p is not a substrate for Plk1 *in* *vitro* (Fig. 8), strongly implying that the physical and genetic interactions described above are likely to have a functional role. Defining the phospho-acceptor sites will facilitate future studies in this area.

Finally, we have shown that the physical interactions observed in fission yeast are recapitulated in human cells (Fig. 8), providing the intriguing suggestion that Plk1 may regulate ESCRT function in higher eukaryotes; such regulatory mechanisms have already been shown for Aurora B kinase-mediated control of abscission timing in human cells [8]. Hence, regulation by Plk1 may provide a further layer of control of this process. ESCRT phosphorylation status may elicit conformational changes that drive abscission, such as ESCRT-III nucleation [5]. Consistent with this model, Plk1 has recently been shown to reappear at the midbody of human cells very late in telophase, a time when the ESCRT complex assembles at the abscission site [10]. Therefore, future characterisation of these interactions and their functional significances may provide useful insights towards manipulating ESCRT proteins in controlling cell division in disease processes.

In summary, our study has shown that ESCRT proteins play a role in septation and cell separation in fission yeast; the interactions observed in this study have been summarised in Table S3 in File S1. Genetic interactions were observed between ESCRT genes and *plo1^+^*, *ark1^+^* and *clp1* ^+^. Furthermore, novel roles were demonstrated for Plo1p, Ark1p and Clp1p in vacuolar sorting in fission yeast, and genetic analysis facilitated characterisation of a genetic pathway in vacuolar sorting for *plo1^+^*, ESCRT genes and *clp1^+^*. Yeast two-hybrid analysis of fission yeast proteins revealed physical interactions between Plo1p and ESCRT proteins from classes 0, I, II and III, interactions which also appear to be present within human cells. Finally, we show that human Plk1 can phosphorylate budding yeast ESCRT-III complex proteins *in* *vitro*, providing evidence that the physical interactions may constitute a conserved control node for ESCRT function. Further characterisation of these interactions and their functional significances will provide useful insights into the regulation of ESCRT proteins in a range of cellular events, including the control of cell division in disease processes.

## Supporting Information

Figure S1 **ESCRT proteins are required for septation in fission yeast.** Wild-type and strains containing individual chromosomal deletions of ESCRT genes were grown at 30°C in complete liquid medium to mid-exponential phase and harvested. Cells were stained with Calcofluor white and visualised using fluorescence microscopy. Images were captured of both fluorescence and bright field. Scale bar, 10 µm. The frequency of phenotypes A–F (described in Fig. 1a) was quantitatively analysed in strains containing double mutants, in comparison to wild-type. In each case 400 cells were counted in triplicate (*p<0.05; n = 3). Each of the ESCRT genes labels is accompanied by its respective ESCRT complex identification (*E-0*, *E-I*, *E-II* and *E-III*).
(TIF)

Figure S2 ***plo1-ts35*** **shows synthetic growth phenotypes with** ***vps28Δ*** **.** Single and double mutants of *plo1-ts35* and *vps28Δ* were grown in liquid YE at 30°C. Cultures were spotted on solid YE to the orders of cell density indicated and images were captured after three days of growth at 30°C.
(TIF)

Figure S3 **Plo1p, Ark1 and Clp1 are required for septation in fission yeast.** Wild-type and strains containing mutations in *plo1*, *ark1* or *clp1* genes were grown at 25°C in complete liquid medium to mid-exponential phase and harvested. Cells were stained with Calcofluor white and visualised using fluorescence microscopy. Both fluorescence and bright field images are shown. Scale bar, 10 µm. The frequency of phenotypes A–F (described in Fig. 1a) was quantitatively analysed in strains containing double mutants, in comparison to wild-type. In each case 400 cells were counted in triplicate (*p<0.05; n = 3). Each of the ESCRT genes labels is accompanied by its respective ESCRT complex identification (*E-0*, *E-I*, *E-II* and *E-III*).
(TIF)

Figure S4 **Synthetic cytokinetic phenotypes observed in double mutants in genes encoding ESCRT proteins and** ***ark1-T11*** **.** Fission yeast double mutant strains were grown in complete liquid medium at 25°C to mid-exponential phase and harvested. Cells were stained with Calcofluor white and visualised using fluorescence microscopy. Images were captured of both fluorescence and bright field. Scale bar, 10 µm. The frequency of phenotypes A–F (described in Fig. 1a) was quantitatively analysed in strains containing double mutants, in comparison to each parent. In each case 400 cells were counted in triplicate (*p<0.05; n = 3). Each of the ESCRT genes labels is accompanied by its respective ESCRT complex identification (*E-0*, *E-I*, *E-II* and *E-III*).
(TIF)

Figure S5 **Over-expressed CHMP6-GFP co-immunoprecipitates with Plk1 from HEK293 cell lysates.** (a) HEK293 cells were transfected with DNA for CHMP6-GFP or FIP3-GFP. Lysates were prepared and anti-Plk1 antibody was added to each cell lysate. (b) CHMP6-GFP or with Lipofectamine 2000 in the absence of transforming DNA were added to cells, lysates were prepared and anti-Plk1 antibody was added to each cell lysate. The lysate following incubation with Protein A sepharose beads was retained (*supernatant* in figure: 5 µg was loaded). Anti-Plk1 antibody was incubated in the supernatant (S1). Centrifugation following incubation with Protein A sepharose beads resulted in S2. This was interrogated for a third time with anti-Plk1 antibody and beads, resulting in S3. A Protein A sepharose beads-only immunoprecipitation was performed on S3, resulting in S4. Non-transfected lysates were also interrogated with anti-Plk1 antibody. Complexes were dissociated from beads (*IP*: one-fifteenth was loaded). *Input* refers to the cell lysate that was interrogated: 5 µg was loaded. Experiments were repeated with qualitatively similar results.
(TIF)

File S1 **Table S1, Genetic interactions between mutants of ESCRT genes with mutants of** ***plo1*** **,** ***ark1*** **, or** ***clp1*** **.** Table S2, Summary of epistatic interactions observed between genes encoding ESCRT proteins, polo and aurora kinase and Cdc14 phosphatase. Table S3, Summary of interactions observed between ESCRT proteins, polo and aurora kinases and Cdc14 phosphatase. Table S4, Fission yeast strains used in this study. Table S5, Budding yeast strains used in this study.
(DOCX)
