# Supplementary figures and images for: A Complex Network of Interactions between Mitotic Kinases, Phosphatases and ESCRT Proteins Regulates Septation and Membrane Trafficking in S. pombe
Source: PLoS One. 2014 Oct 30;9(10):e111789. doi: 10.1371/journal.pone.0111789 (PMC4214795; doi:10.1371/journal.pone.0111789)

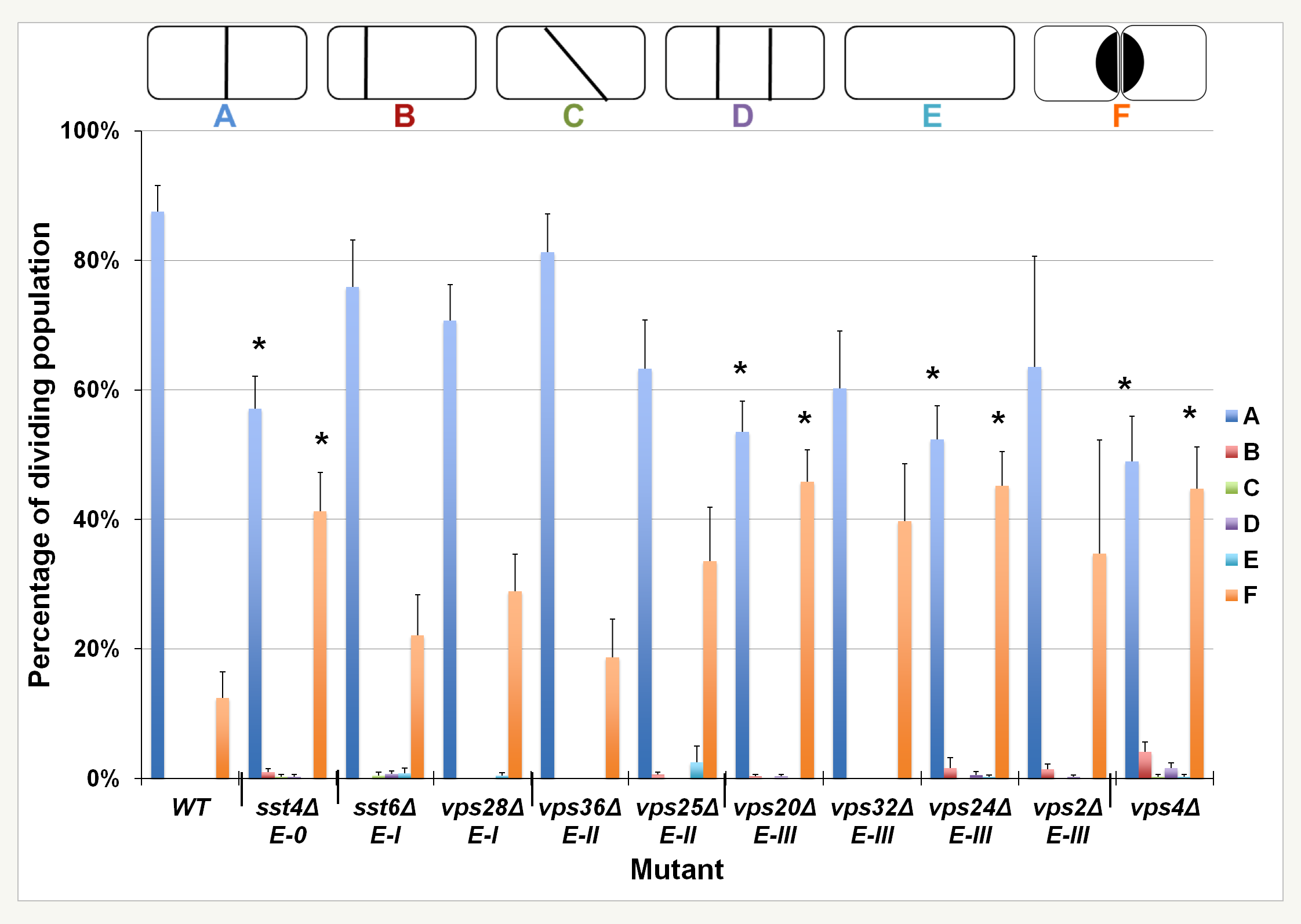

Supplement: Figure S1 — ESCRT proteins are required for septation in fission yeast. Wild-type and strains containing individual chromosomal deletions of ESCRT genes were grown at 30°C in complete liquid medium to mid-exponential phase and harvested. Cells were stained with Calcofluor white and visualised using fluorescence microscopy. Images were captured of both fluorescence and bright field. Scale bar, 10 µm. The frequency of phenotypes A–F (described in Fig. 1a) was quantitatively analysed in strains containing double mutants, in comparison to wild-type. In each case 400 cells were counted in triplicate (*p<0.05; n = 3). Each of the ESCRT genes labels is accompanied by its respective ESCRT complex identification (E-0, E-I, E-II and E-III). (TIF) [file pone.0111789.s001.tif]

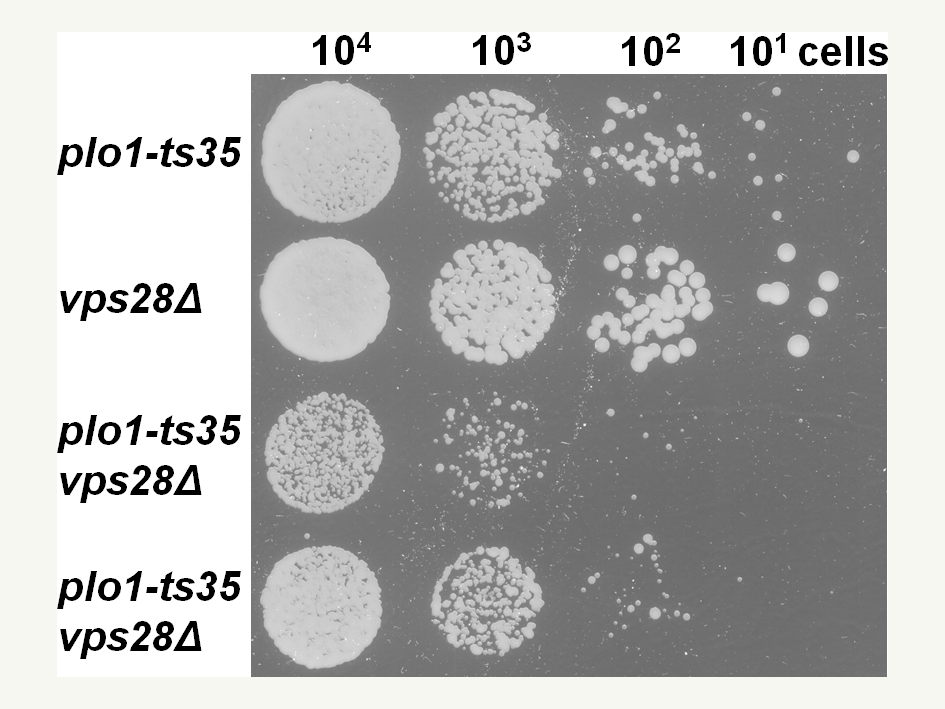

Supplement: Figure S2 — plo1-ts35 shows synthetic growth phenotypes with vps28Δ . Single and double mutants of plo1-ts35 and vps28Δ were grown in liquid YE at 30°C. Cultures were spotted on solid YE to the orders of cell density indicated and images were captured after three days of growth at 30°C. (TIF) [file pone.0111789.s002.tif]

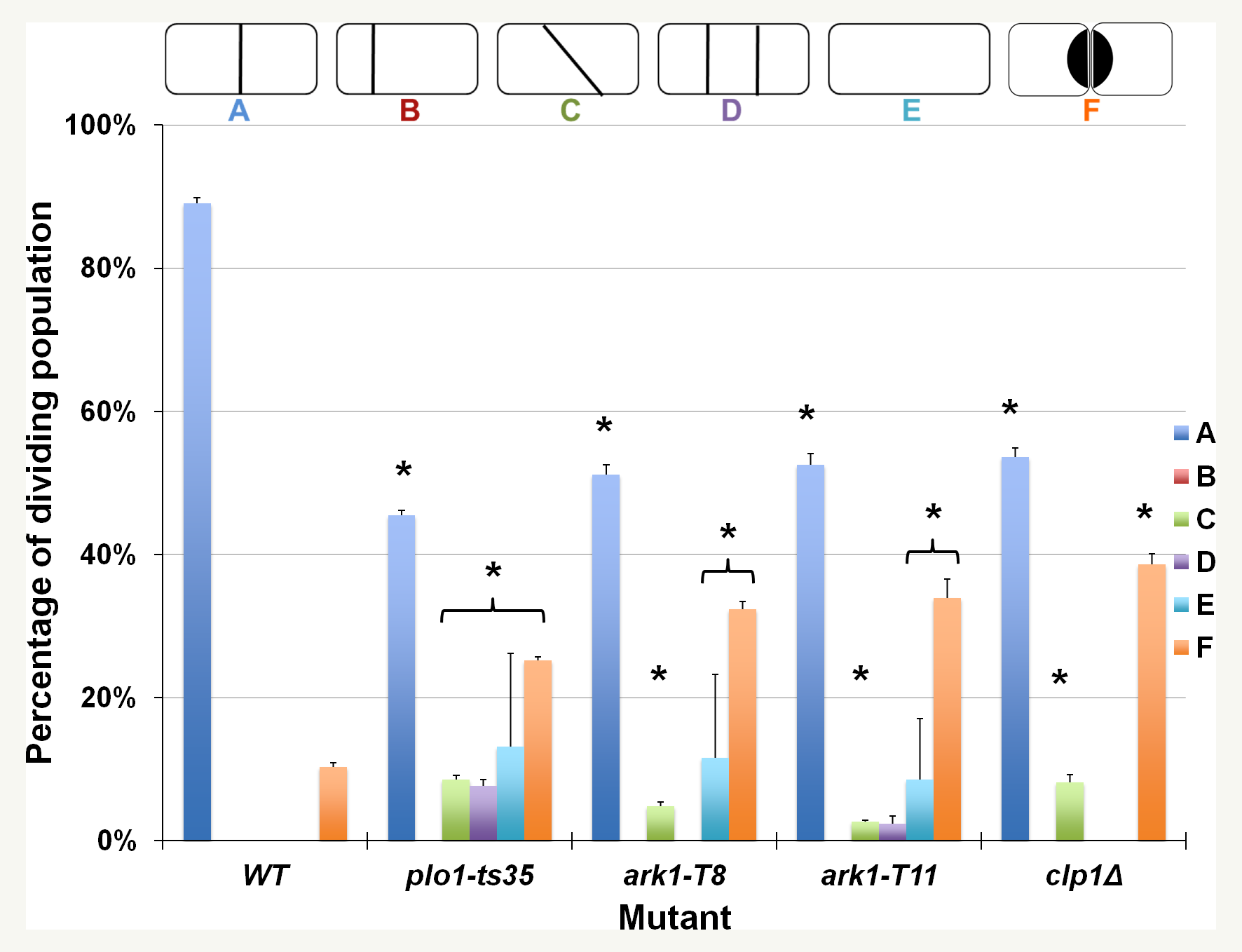

Supplement: Figure S3 — Plo1p, Ark1 and Clp1 are required for septation in fission yeast. Wild-type and strains containing mutations in plo1, ark1 or clp1 genes were grown at 25°C in complete liquid medium to mid-exponential phase and harvested. Cells were stained with Calcofluor white and visualised using fluorescence microscopy. Both fluorescence and bright field images are shown. Scale bar, 10 µm. The frequency of phenotypes A–F (described in Fig. 1a) was quantitatively analysed in strains containing double mutants, in comparison to wild-type. In each case 400 cells were counted in triplicate (*p<0.05; n = 3). Each of the ESCRT genes labels is accompanied by its respective ESCRT complex identification (E-0, E-I, E-II and E-III). (TIF) [file pone.0111789.s003.tif]

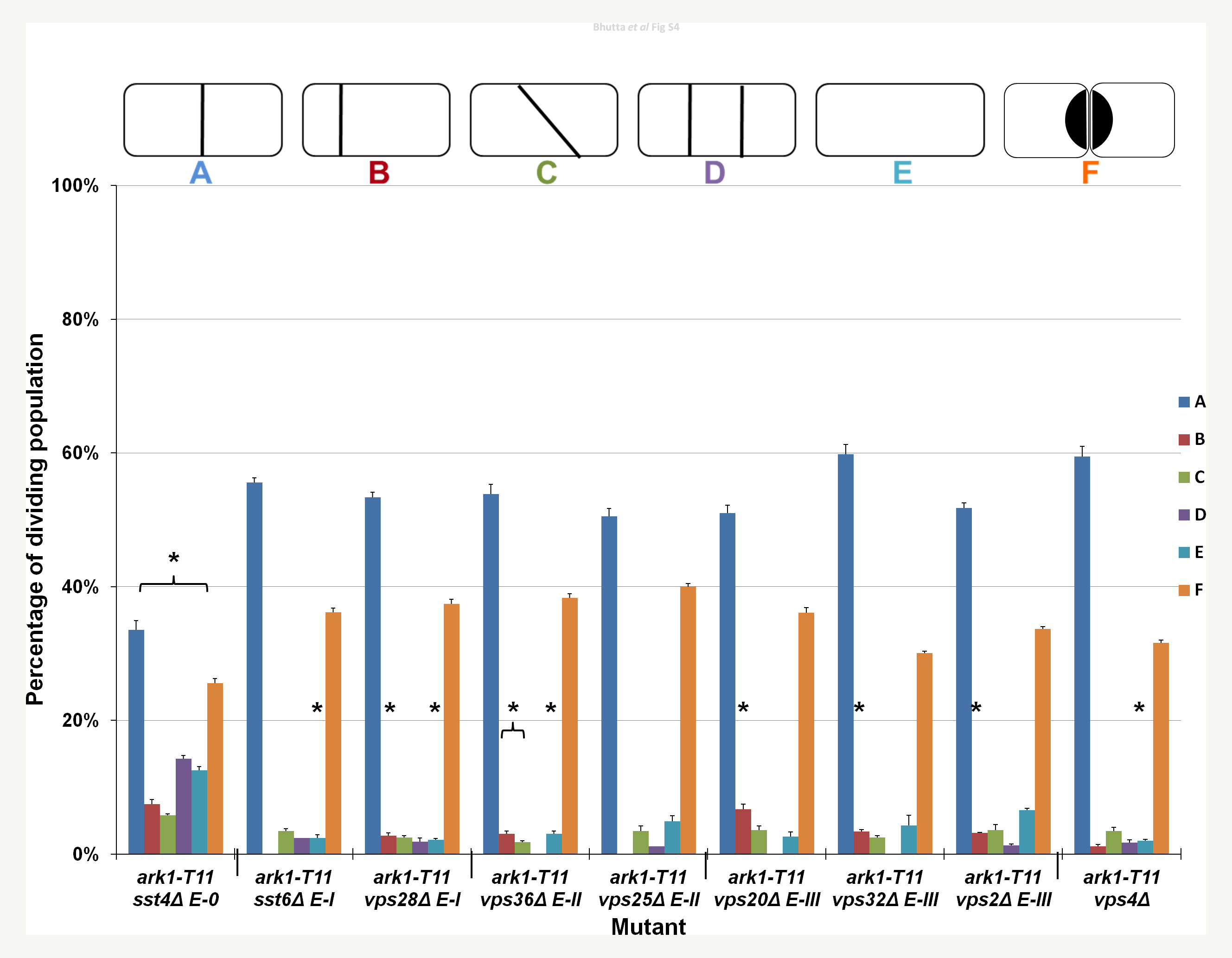

Supplement: Figure S4 — Synthetic cytokinetic phenotypes observed in double mutants in genes encoding ESCRT proteins and ark1-T11 . Fission yeast double mutant strains were grown in complete liquid medium at 25°C to mid-exponential phase and harvested. Cells were stained with Calcofluor white and visualised using fluorescence microscopy. Images were captured of both fluorescence and bright field. Scale bar, 10 µm. The frequency of phenotypes A–F (described in Fig. 1a) was quantitatively analysed in strains containing double mutants, in comparison to each parent. In each case 400 cells were counted in triplicate (*p<0.05; n = 3). Each of the ESCRT genes labels is accompanied by its respective ESCRT complex identification (E-0, E-I, E-II and E-III). (TIF) [file pone.0111789.s004.tif]

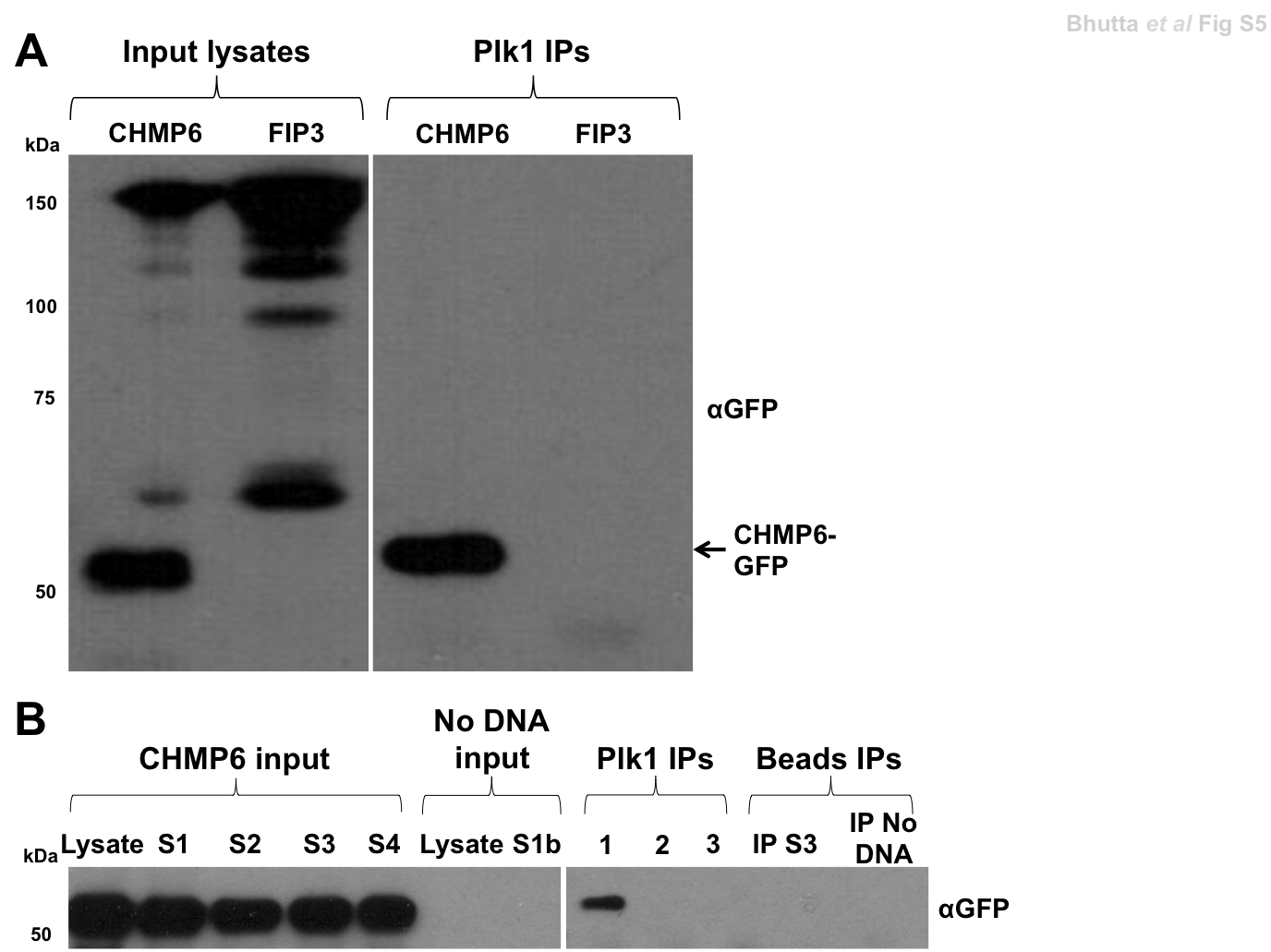

Supplement: Figure S5 — Over-expressed CHMP6-GFP co-immunoprecipitates with Plk1 from HEK293 cell lysates. (a) HEK293 cells were transfected with DNA for CHMP6-GFP or FIP3-GFP. Lysates were prepared and anti-Plk1 antibody was added to each cell lysate. (b) CHMP6-GFP or with Lipofectamine 2000 in the absence of transforming DNA were added to cells, lysates were prepared and anti-Plk1 antibody was added to each cell lysate. The lysate following incubation with Protein A sepharose beads was retained (supernatant in figure: 5 µg was loaded). Anti-Plk1 antibody was incubated in the supernatant (S1). Centrifugation following incubation with Protein A sepharose beads resulted in S2. This was interrogated for a third time with anti-Plk1 antibody and beads, resulting in S3. A Protein A sepharose beads-only immunoprecipitation was performed on S3, resulting in S4. Non-transfected lysates were also interrogated with anti-Plk1 antibody. Complexes were dissociated from beads (IP: one-fifteenth was loaded). Input refers to the cell lysate that was interrogated: 5 µg was loaded. Experiments were repeated with qualitatively similar results. (TIF) [file pone.0111789.s005.tif]
