# Supplementary material for: A Complex Network of Interactions between Mitotic Kinases, Phosphatases and ESCRT Proteins Regulates Septation and Membrane Trafficking in S. pombe
Source: PLoS One. 2014 Oct 30;9(10):e111789. doi: 10.1371/journal.pone.0111789 (PMC4214795; doi:10.1371/journal.pone.0111789)
Supplement: File S1 — Table S1, Genetic interactions between mutants of ESCRT genes with mutants of plo1 , ark1 , or clp1 . Table S2, Summary of epistatic interactions observed between genes encoding ESCRT proteins, polo and aurora kinase and Cdc14 phosphatase. Table S3, Summary of interactions observed between ESCRT proteins, polo and aurora kinases and Cdc14 phosphatase. Table S4, Fission yeast strains used in this study. Table S5, Budding yeast strains used in this study. (DOCX) [file pone.0111789.s006.docx]

**SUPPLEMENTARY INFORMATION**

**A complex network of interactions between mitotic kinases, phosphatases and ESCRT proteins regulates septation and membrane trafficking in *S. pombe***

Musab S. Bhutta, Brinta Roy, Gwyn W. Gould and Christopher J. McInerny

Henry Wellcome Laboratory of Cell Biology,

Davidson Building,

Institute of Molecular, Cell and Systems Biology,

College of Medical, Veterinary and Life Sciences,

University of Glasgow,

Glasgow G12 8QQ

United Kingdom

Correspondence to:

e: [Chris.McInerny@Glasgow.ac.uk](mailto:Chris.McInerny@Glasgow.ac.uk) or Gwyn.Gould@Glasgow.ac.uk

t: 0141 330 3208 (CJM) or 0141 330 5263 (GWG)

**Table S1. Genetic interactions between mutants of ESCRT genes with mutants of *plo1*, *ark1*, or *clp1*.**

| ***ESCRTΔ*** | ***plo1-ts35*** | ***ark1-T8*** | ***clp1Δ*** | ***clp1.D257A*** | ***clp1.3A*** |
| --- | --- | --- | --- | --- | --- |
| ***sst4Δ E-0*** | **✓** | **✓** | **✓** | **✓** | **✗** |
| ***sst6Δ E-I*** | **✓** | **✗** | ***Tight linkage of genes on chromosome precludes analysis*** | | |
| ***vps28Δ E-I*** | **✓** | **✗** | **✓** | **✗** | **✓** |
| ***vps36Δ E-II*** | **✗** | **✓** | **✓** | **✗** | **✓** |
| ***vps25Δ E-II*** | **✓** | **✗** | **✗** | **✗** | **✗** |
| ***vps20Δ E-III*** | **✓** | **✓** | **✓** | **✓** | **✓** |
| ***vps2Δ E-III*** | **✓** | **✗** | **✓** | **✗** | **✗** |
| ***vps4Δ*** | **✓** | **✓** | **✓** | **✓** | **✓** |

Double mutants that resulted in synthetic growth phenotypes, specifically slower growth rates at particular temperatures, are indicated by ticks.

**Table S2. Summary of epistatic interactions observed between genes encoding ESCRT proteins*,* polo and aurora kinase and Cdc14 phosphatase.**

| ***ESCRTΔ*** | ***plo1-ts35*** | ***ark1-T8*** | ***clp1Δ*** |
| --- | --- | --- | --- |
| ***sst4Δ E-0*** | *plo1-ts35* | *ark1-T8/sst4Δ* | *sst4Δ* |
| ***vps28Δ E-I*** | *plo1-ts35* | *ark1-T8/vps28Δ* | *vps28Δ* |
| ***vps36Δ E-II*** | *plo1-ts35* | *ark1-T8/vps36Δ* | *vps36Δ* |
| ***vps20Δ E-III*** | *plo1-ts35* | *ark1-T8/vps20Δ* | *vps20Δ* |
| ***vps4Δ*** | *vps4Δ* | *ark1-T8/vps4Δ* | *vps4Δ* |

**Table S3. Summary of interactions observed between ESCRT proteins*,* polo and aurora kinases and Cdc14 phosphatase.**


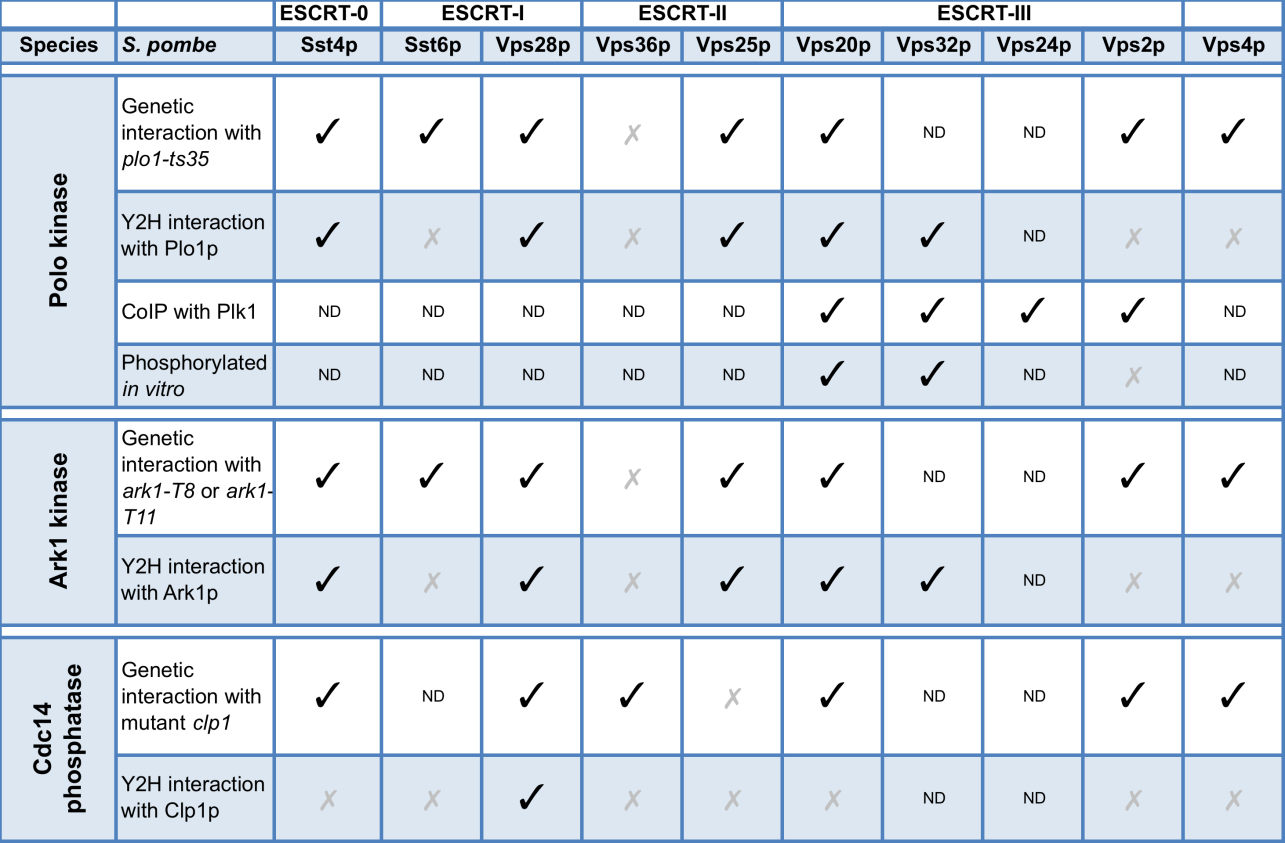


Y2H, yeast two-hybrid; CoIP, co-immunoprecipitation; ND, not determined.

**Table S4. Fission yeast strains used in this study.**

Collection Genotype ESCRT CLASS Source

GG 1 h^-^ 972 (wild-type) Lab stock

GG 308 h^-^ *cdc25-22* Lab stock

GG 354 h^-^ *plo1-ts35* Anderson et al. 2002

GG 1146 h^+^ *clp1*::*ura4*^+^ Lab stock

GG 1181 h^-^ *clp1.D257A-13myc:kanR ade6-* Lab stock

GG 1182 h^-^ *clp1.3A-13myc:kanR ade6-* Lab stock

GG 1307 h^+^ *clp1*::*kanR* Lab stock

GG 2412 h^+^ *ark1-T11<<kanR*  This study

GG 2432 h^-^ *ark1-T8-GFP-flag-His<<kanR* This study

GG 1604 h^90^ *sst4*::*ura4*^+^  E0  Iwaki et al. 2007

GG 1596 h^90^ *sst6*::*ura4*^+^ *ade6-* EI Iwaki et al. 2007

GG 1597 h^90^ *vps28*::*ura4*^+^ *ade6-* EI Iwaki et al. 2007

GG 1598 h^90^ *vps36*::*ura4*^+^ EII Iwaki et al. 2007

GG 1599 h^90^ *vps25*::*ura4*^+^ EII Iwaki et al. 2007

GG 1600 h^90^ *vps20*::*ura4*^+^ *ade6-* EIII Iwaki et al. 2007

GG 2531 h90 *vps32*:*ura4*+ *ade6-* EIII Iwaki et al. 2007

GG 1600 h90 *vps24*::*ura4*+ *ade6-* EIII Iwaki et al. 2007

GG 1602 h^-^ *vps2*::*LEU2* EIII Iwaki et al. 2007

GG 1603 h^90^ *vps4*::*ura4*^+^ Iwaki et al. 2007

GG 1666 h^+^ *sst4*::*ura4*^+^ *plo1-ts35* This study

GG 1688 h^90^ *sst6*::*ura4*^+^ *plo1-ts35* This study

GG 1682 h^90^ *vps28*::*ura4*^+^ *plo1-ts35* This study

GG 1676 h^-^ *vps36*::*ura4*^+^ *plo1-ts35* This study

GG 1679 h^+^ *vps25*::*ura4*^+^ *plo1-ts35* This study

GG 1691 h^90^ *vps20*::*ura4*^+^ *plo1-ts35* This study

GG 2559 h^?^ *vps32*::*ura4*^+^ *plo1-ts35* This study

GG 1694 h^+^ *vps2*::*LEU2* *plo1-ts35* This study

GG 1725 h^-^ *vps4*::*ura4*^+^ *plo1-ts35*  This study

GG 2479 h^-^ *sst4*::*ura4*^+^ *ark1-T8-GFP-flag-His<<kanR* This study

GG 2453 h^+^ *sst6*::*ura4*^+^ *ark1-T8-GFP-flag-His<<kanR* This study

GG 2486 h^90^ *vps28*::*ura4*^+^ *ark1-T8-GFP-flag-His<<kanR* This study

GG 2496 h^-^ *vps36*::*ura4*^+^ *ark1-T8-GFP-flag-His<<kanR* This study

GG 2506 h^+^ *vps25*::*ura4*^+^ *ark1-T8-GFP-flag-His<<kanR* This study

GG 2518 h^+^ *vps20*::*ura4*^+^ *ark1-T8-GFP-flag-His<<kanR* This study

GG 2575 h^?^ *vps32*:*ura4*^+^ *ark1-T8-GFP-flag-His<<kanR* This study

GG 2530 h^+^ *vps2*::*LEU2* *ark1-T8-GFP-flag-His<<kanR* This study

GG 2466 h^-^ *vps4*::*ura4*^+^ *ark1-T8-GFP-flag-His<<kanR* This study

GG 2485 h^-^ *sst4*::*ura4*^+^ *ark1-T11-GFP-flag-His<<kanR* This study

GG 2458 h^+^ *sst6*::*ura4*^+^ *ark1-T11<<kanR* This study

GG 2491 h^+^ *vps28*::*ura4*^+^ *ark1-T11<<kanR* This study

GG 2501 h^+^ *vps36*::*ura4*^+^ *ark1-T11<<kanR* This study

GG 2511 h^-^ *vps25*::*ura4*^+^ *ark1-T11<<kanR* This study

GG 2522 h^+^ *vps20*::*ura4*^+^ *ark1-T11<<kanR* This study

GG 2566 h^?^ *vps32*:*ura4*^+^ *ark1-T11<<kanR* This study

GG 2423 h^-^ *vps2*::*LEU2* *ark1-T11<<kanR* This study

GG 2471 h^-^ *vps4*::*ura4*^+^ *ark1-T11-GFP-flag-His<<kanR* This study

GG 1660 h^+^ *sst4*::*ura4*^+^ *clp1*::*kanR* This study

GG 1637 h^90^ *vps28*::*ura4*^+^ *clp1*::*kanR* This study

GG 1654 h^+^ *vps36*::*ura4*^+^ *clp1*::*kanR* This study

GG 1657 h^-^ *vps25*::*ura4*^+^ *clp1*::*kanR* This study

GG 1649 h^90^ *vps20*::*ura4*^+^ *clp1*::*kanR* This study

GG 2562 h^?^ *vps32*::*ura4*^+^ *clp1*::*kanR* This study

GG 1646 h^+^ *vps2*::*LEU2* *clp1*::*kanR* This study

GG 1663 h^+^ *vps4*::*ura4*^+^ *clp1*::*kanR* This study

GG 1706 h^-^ *sst4*::*ura4*^+^ *clp1.D257A-13myc:kanR* This study

GG 1717 h^90^ *vps28*::*ura4*^+^ *clp1.D257A-13myc:kanR* This study

GG 1760 h^+^ *vps36*::*ura4*^+^ *clp1.D257A-13myc:kanR* This study

GG 1765 h^-^ *vps25*::*ura4*^+^ *clp1.D257A-13myc:kanR* This study

GG 1785 h^90^ *vps20*::*ura4*^+^ *clp1.D257A-13myc:kanR* This study

GG 1791 h^-^ *vps2*::*LEU2* *clp1.D257A-13myc:kanR* This study

GG1842 h^?^ *sst4*::*ura4*^+^ *clp1.3A-13myc:kanR* This study

GG 1805 h^?^ *vps28*::*ura4*^+^ *clp1.3A-13myc:kanR* This study

GG 1809 h^?^ *vps36*::*ura4*^+^ *clp1.3A-13myc:kanR* This study

GG 1826 h^?^ *vps25*::*ura4*^+^ *clp1.3A-13myc:kanR* This study

GG1844 h^?^ *vps20*::*ura4*^+^ *clp1.3A-13myc:kanR* This study

GG1850 h^?^ *vps2*::*LEU2* *clp1.3A-13myc:kanR* This study

GG1839 h^?^ *vps4*::*ura4*^+^ *clp1.3A-13myc:kanR cdc25-22* This study

GG 2127 pREP41:Ub-GFP-SpCPS

h^-^ This study

GG 2184 pREP41:Ub-GFP-SpCPS

h^-^ *sst4*::*ura4*^+^ This study

GG 2139 pREP41:Ub-GFP-SpCPS

h^+^ *sst6*::*ura4*^+^ This study

GG 2187 pREP41:Ub-GFP-SpCPS

h^90^ *vps28*::*ura4*^+^ This study

GG 2190 pREP41:Ub-GFP-SpCPS

h^-^ *vps36*::*ura4*^+^ This study

GG 2142 pREP41:Ub-GFP-SpCPS

h^+^ *vps20:*:*ura4*^+^ This study

GG 2193 pREP41:Ub-GFP-SpCPS

h^-^ *vps4*::*ura4*^+^ This study

GG 2206 pREP41:Ub-GFP-SpCPS

*plo1-ts35* This study

GG 2632 pREP41:Ub-GFP-SpCPS

h^+^ *ark1-T11<<kanR leu1-32* This study

GG 2642 pREP41:Ub-GFP-SpCPS

h^+^ *ark1-T8-GFP-flag-His<<kanR leu1-32* This study

GG 2196 pREP41:Ub-GFP-SpCPS

h^+^ *clp1*::*ura4*^+^ This study

GG 2275 pREP41:Ub-GFP-SpCPS

h^-^ *clp1.3A-13myc:kanR* This study

GG 2302 pREP41:Ub-GFP-SpCPS

h^-^ *clp1.D257A-13myc:kanR* This study

GG 2239 pREP41:Ub-GFP-SpCPS

h^+^ *sst4*::*ura4*^+^ *plo1-ts35* This study

GG 2242 pREP41:Ub-GFP-SpCPS

h^90^ *vps28*::*ura4*^+^ *plo1-ts35* This study

GG 2245 pREP41:Ub-GFP-SpCPS

h^90^ *vps20*::*ura4*^+^ *plo1-ts35* This study

GG 2248 pREP41:Ub-GFP-SpCPS

h^-^ *vps4*::*ura4*^+^ *plo1-ts35* This study

GG 2251 pREP41:Ub-GFP-SpCPS

h^-^ *vps36*::*ura4*^+^ *plo1-ts35* This study

GG 2634 pREP41:Ub-GFP-SpCPS

h^+^ *sst4*::*ura4*^+^ *ark1-T8-GFP-flag-His<<kanR*  This study

GG 2652 pREP41:Ub-GFP-SpCPS

h^+^ *vps28*::*ura4*^+^ *ark1-T8-GFP-flag-His<<kanR* This study

GG 2648 pREP41:Ub-GFP-SpCPS

h^+^ *vps36*::*ura4*^+^ *ark1-T8-GFP-flag-His<<kanR* This study

GG 2640 pREP41:Ub-GFP-SpCPS

h^+^ *vps20*::*ura4*^+^ *ark1-T8-GFP-flag-His<<kanR* This study

GG 2646 pREP41:Ub-GFP-SpCPS

h^-^ *vps4*::*ura4*^+^ *ark1-T8-GFP-flag-His<<kanR* This study

GG 2216 pREP41:Ub-GFP-SpCPS

h^+^ *sst4*::*ura4*^+^ *clp1*::*kanR* This study

GG 2219 pREP41:Ub-GFP-SpCPS

h^90^ *vps28*::*ura4*^+^ *clp1*::*kanR* This study

GG 2222 pREP41:Ub-GFP-SpCPS

h^+^ *vps36*::*ura4*^+^ *clp1*::*kanR* This study

GG 2225 pREP41:Ub-GFP-SpCPS

h^90^ *vps20*::*ura4*^+^ *clp1*::*kanR* This study

GG 2228 pREP41:Ub-GFP-SpCPS

h^+^ *vps4*::*ura4*^+^ *clp1*::*kanR* This study

All were *leu1-32 ura4-D18* unless otherwise stated. *ade6-* is either *ade6-M210* or *ade6-M216.*

**Table S5. Budding yeast strains used in this study.**

Collection Genotype Source

GGBY 138 MATa *URA3*::*lexA-lacZ met- his3 ade2 trp1 leu2*

*gal4Δ gal80Δ*  Lab stock

GGBY 171 MATa *trp1-190 leu2-3, 112, ura 3-52 his 3-200* Lab stock *gal4Δ gal80Δ*

*LYS2::GAL1-HIS3, GAL2-ADE2 met2::GAL-lacZ*
